# Pulmonary toxicity and translocation of gallium phosphide nanowires to secondary organs following pulmonary exposure in mice

**DOI:** 10.1186/s12951-023-02049-0

**Published:** 2023-09-07

**Authors:** Trine Berthing, Mercy Lard, Pernille H. Danielsen, Laura Abariute, Kenneth K. Barfod, Karl Adolfsson, Kristina B. Knudsen, Henrik Wolff, Christelle N. Prinz, Ulla Vogel

**Affiliations:** 1 The National Research Centre for the Working Environment, Copenhagen, Denmark; 2 Division of Solid State Physics and NanoLund, Lund University, Lund, 22 100 Sweden; 3 Finnish Institute of Occupational Health, Helsinki, Finland; 4 Present Address: Phase Holographic Imaging PHI AB, Lund, 224 78 Sweden; 5 Present Address: Department of Food Science, Microbiology and Fermentation, University of Copenhagen, Copenhagen, Denmark; 6 Present Address: Axis Communications AB, Lund, 223 69 Sweden; 7 Department of Pathology, University of Helsinki, Helsinki, Finland; 8 National Food Institute, Technical University of Denmark, Kgs. Lyngby, Denmark

**Keywords:** Nanowires, Pulmonary exposure, Inflammation, Biodistribution, High aspect ratio nanomaterial (HARN)

## Abstract

**Background:**

III-V semiconductor nanowires are envisioned as being integrated in optoelectronic devices in the near future. However, the perspective of mass production of these nanowires raises concern for human safety due to their asbestos- and carbon nanotube-like properties, including their high aspect ratio shape. Indeed, III-V nanowires have similar dimensions as Mitsui-7 multi-walled carbon nanotubes, which induce lung cancer by inhalation in rats. It is therefore urgent to investigate the toxicological effects following lung exposure to III-V nanowires prior to their use in industrial production, which entails risk of human exposure. Here, female C57BL/6J mice were exposed to 2, 6, and 18 µg (0.12, 0.35 and 1.1 mg/kg bw) of gallium phosphide (III-V) nanowires (99 nm diameter, 3.7 μm length) by intratracheal instillation and the toxicity was investigated 1, 3, 28 days and 3 months after exposure. Mitsui-7 multi-walled carbon nanotubes and carbon black Printex 90 nanoparticles were used as benchmark nanomaterials.

**Results:**

Gallium phosphide nanowires induced genotoxicity in bronchoalveolar lavage cells and acute inflammation with eosinophilia observable both in bronchoalveolar lavage and lung tissue (1 and 3 days post-exposure). The inflammatory response was comparable to the response following exposure to Mitsui-7 multi-walled carbon nanotubes at similar dose levels. The nanowires underwent partial dissolution in the lung resulting in thinner nanowires, with an estimated in vivo half-life of 3 months. Despite the partial dissolution, nanowires were detected in lung, liver, spleen, kidney, uterus and brain 3 months after exposure.

**Conclusion:**

Pulmonary exposure to gallium phosphide nanowires caused similar toxicological effects as the multi-walled carbon nanotube Mitsui-7.

**Graphical Abstract:**

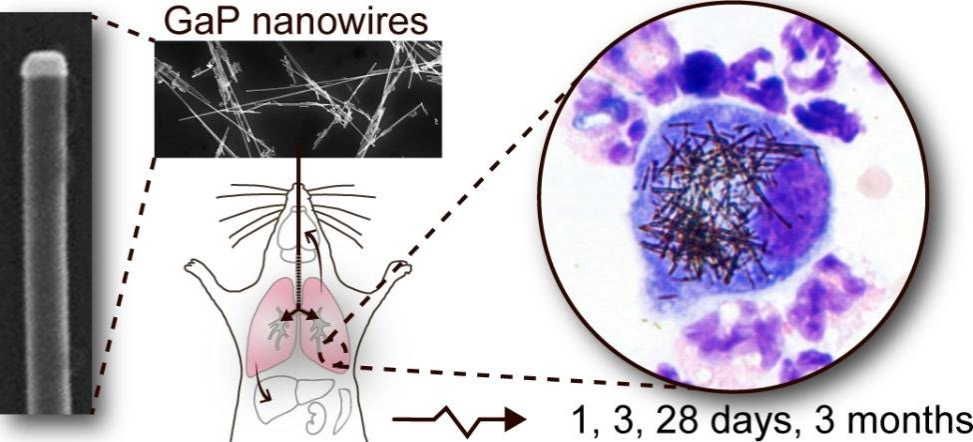

**Supplementary Information:**

The online version contains supplementary material available at 10.1186/s12951-023-02049-0.

## Background

III-V semiconductor nanowires (NWs) have a great potential to improve current optoelectronic devices. For instance, researchers envision that III-V NWs, because of their small size and high electron mobility, may replace current silicon-based transistors in the future [1]. With a high surface-to-projected-area ratio and tunable bandgaps, vertical arrays of III-V NW solar cells are expected to improve solar panel efficiencies dramatically [2]. III-V NWs are high aspect ratio nanomaterials, typically synthesized from metallic seed particles using epitaxy [3]. Their geometry can be controlled to a high degree: their diameter generally ranges from 10 to 200 nm, and their length from 1 to 20 μm. However, from a human health perspective, III-V NWs are of great concern. Indeed, many of the III-V elements such as arsenic and antimony are toxic if released in the human body following inhalation and pulmonary deposition [4]. On the other hand, if the NWs are insoluble in lung tissue, NWs may comply with the fiber pathogenicity paradigm that causally links inhalation of bio-persistent fibers to severe lung diseases and cancer [5]. If III-V NWs emerge as key components in future electronic and photovoltaic devices, their production will move from laboratory custom synthesis to mass production. Therefore, it is crucial to assess the potential adverse health effects of III-V NW exposure to ensure human safety in the further development of this promising technology.

In terms of biological interactions with III-V NWs in vivo or in vitro, most of the studies found in the literature describe in vitro studies of living cell properties on vertical arrays of NWs [6–12]. Some applications using NWs for biosensing are also described [13–18]. However, fewer studies have investigated the effects of III-V NWs on whole organisms. We have investigated the effects of NWs following injection into the rat brain. Although this research was designed in the context of developing brain implants, it confirmed that III-V NWs comply with the fiber pathogenicity paradigm [19, 20]: insoluble NWs that were longer than the size of immune cells (5 μm for rat brain) elicit a long-lasting inflammation in the brain, whereas slowly dissolvable and/or shorter NWs only affect the brain tissue transiently. We have also investigated the effects of NWs in *Drosophila melanogaster* and *Daphnia magna* using ingestion as exposure route [21, 22], which is not the most relevant exposure route for humans. The most likely human exposure route for III-V nanowires and engineered nanomaterials, especially in the work environment, is inhalation [23, 24]. Whereas small-scale synthesis of NWs is usually carried out as growth from substrates, large-scale synthesis of NWs is expected to be performed using aerotaxy, where NWs are synthesized from gasses resulting in aerosolized fibers and consequently, an increased risk of inhalation exposure is anticipated [25]. Furthermore, inhalation exposure may occur during waste treatment. Inhalation of high aspect ratio nanomaterials has been proposed to induce lung cancer due to the adherence to the fiber pathogenicity paradigm. Recently, one specific type of multi-walled carbon nanotube (MWCNT), called MWNT-7 or Mitsui-7, was classified as possibly carcinogenic to humans by the International Agency for Research on Cancer [26]. This specific carbon nanotube caused dose-dependent lung cancer in a two-year inhalation study in rats [27]. Low occupational exposure limits for MWCNTs (0.001 mg/m^3^) have been proposed by the National Institute for Occupational Safety & Health (NIOSH) and others [28, 29]. The European Chemicals Agency (ECHA) recently proposed to classify multi-walled carbon nanotubes (MWCNTs) as category 1B carcinogens if they have a geometric tube diameter between 30 nm and 3 μm, a length above 5 μm and an aspect ratio above 3:1 [30]. The fiber-like high aspect ratio geometry of III-V NWs raises the concern that they may have similar toxicological effects as carbon nanotubes following inhalation exposure. Therefore, there is an urgent need for in vivo investigations of the toxicological effects and the fate of III-V NWs that reach the lung. Gallium phosphide (GaP) NWs are a good model system for III-V NWs due to the high degree of control over the GaP NW geometry and due to the relatively high biopersistence of GaP, as compared to NWs composed of other III-V materials or silicon [19, 31–33]. Moreover, GaP NWs have been shown to have a similar Young’s modulus as the one of glass, placing them in the category of stiff fibers [34, 35].

Here, mice were exposed to 2, 6 and 18 µg (0.12, 0.35 and 1.1 mg/kg bw, respectively) of GaP NWs using intratracheal instillation. The toxicity was evaluated at 1, 3, 28 days and 3 months post-exposure, in terms of biodistribution, lung inflammation, genotoxicity and histopathology. For comparison, two benchmark materials were included, carbon black nanoparticles and the MWCNTs Mitsui-7 that have similar physical dimensions to GaP NWs. Moreover, the NW distribution in distant organs was assessed using enhanced darkfield microscopy.

## Results and discussion

This section comprises a rationale for the way the study was designed, characterization of the NWs used in this study, followed by a description of the biological effects.

### Study design

A pilot study was designed based on our previous studies on MWCNT toxicity [36–41]. The dimensions of the GaP NWs used in the pilot study (diameter 78 ± 10 nm, length 1.8 μm ± 3.2 μm) were similar to the dimensions of the MWCNTs NM-401 (67 ± 24 nm diameter, length 4 ± 2.4 μm) and Mitsui-7 (74 ± 28 nm diameter, length 5.7 ± 0.49 μm), which induce inflammation at 6 to 18 µg/mouse [36, 37, 42, 43]. In the pilot study, intratracheal instillation of a single dose level of GaP NWs of 10 µg/mouse was performed. The GaP NW exposure induced acute inflammation in the lungs accompanied by increased numbers of eosinophils observable both in bronchoalveolar lavage at day 3 and lung tissue at day 28. A detailed description of the pilot study is included as supporting information (Table S1 - S2 and Figure S1 - S2). Based on the data from the pilot experiment, a dose-response study with a longer follow-up period was designed using three dose levels of GaP NWs (2, 6 and 18 µg/mouse corresponding to 0.12, 0.35 and 1.1 mg/kg bw, respectively) and four post-exposure time points (1, 3, and 28 days, as well as 3 months after instillation). MWCNT Mitsui-7 (at 6, 18 or 54 µg/mouse, corresponding to 0.32, 0.95 and 2.8 mg/kg bw, respectively) and Printex90 carbon black nanoparticles (at 162 µg/animal corresponding to 9.5 mg/kg bw) were included as benchmark materials.

The occupational exposure levels for NWs in an industrial setting are unknown, therefore it is relevant to compare the chosen dose values to recommendations for carbon nanotubes. For carbon nanotubes, an average exposure level of 0.01 mg/m^3^ was reported for American carbon nanotube production facilities [44], whereas NIOSH has proposed an occupational exposure limit of 0.001 mg/m^3^ [28]. The dose range in the present study of 2 to 18 µg GaP NWs, corresponds to the cumulative pulmonary dose after working 8 months to 5.6 years in an American production facility, or working 6 to 56 years at the proposed occupational exposure limit for carbon nanotubes (assuming a mouse ventilation rate of 1.8 L/h, 10% deposition, a 40 h work week and 45 work weeks per year) [45, 46].

Intratracheal instillation was used as pulmonary exposure method since it allows full control of the deposited dose, which is essential when comparing the toxicity of different materials [47]. In addition, instillation requires much less material compared to inhalation studies. We have previously shown that nanomaterials delivered by intratracheal instillation reach all lung lobes of exposed mice [39, 48]. Furthermore, we and others have shown that inhalation and instillation of MWCNTs, induce qualitatively and quantitatively similar toxicological responses in terms of inflammation, genotoxicity, and perturbed biological pathways [49–53]. More specifically, inhalation and intratracheal exposure to two different MWCNTs induced neutrophil influx in rats that fitted on the same dose-response curve 1–3 days and 1 month after exposure, supporting the use of intratracheal instillation for hazard assessment of nanofibers [49].

### Characterization of GaP NWs

**Fig. 1:**
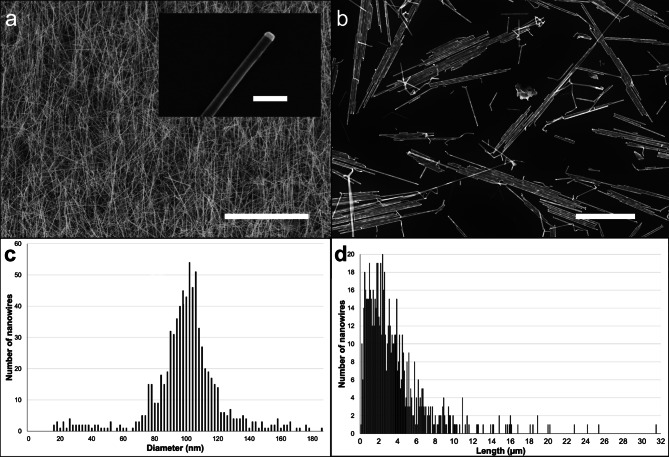
Characterization of GaP NWs. GaP NWs after MOVPE growth **(a)** on the substrate and **(b)** after suspension. SEM images, scale bar **(a)** 20 μm (main), 200 nm (insert), **(b)** 5 μm. GaP NW diameter **(c)** and length **(d)** distribution in suspension

GaP NWs were synthesized using metal-organic vapor-phase epitaxy (MOVPE). The NWs are grown from gold particles that remain on top of the nanowires after MOVPE growth (Fig. 1). The NW diameter is the same as the gold nanoparticle diameter (see insert in Fig. 1a), a feature that was later used to measure dissolution of the NWs. A suspension of NWs for mice instillation was prepared in water with 2% mouse serum (a vehicle previously used for instillation of carbon nanotubes [36, 54]). The average NW diameter was 99 nm ± 24 nm and the average length was 3.7 μm ± 3.6 μm (n = 862 NWs), corresponding to a specific surface area of 9.8 m^2^/g. Of all NWs, 5% were longer than 10 μm.

### Distribution of GaP NWs in lungs of mice

**Fig. 2:**
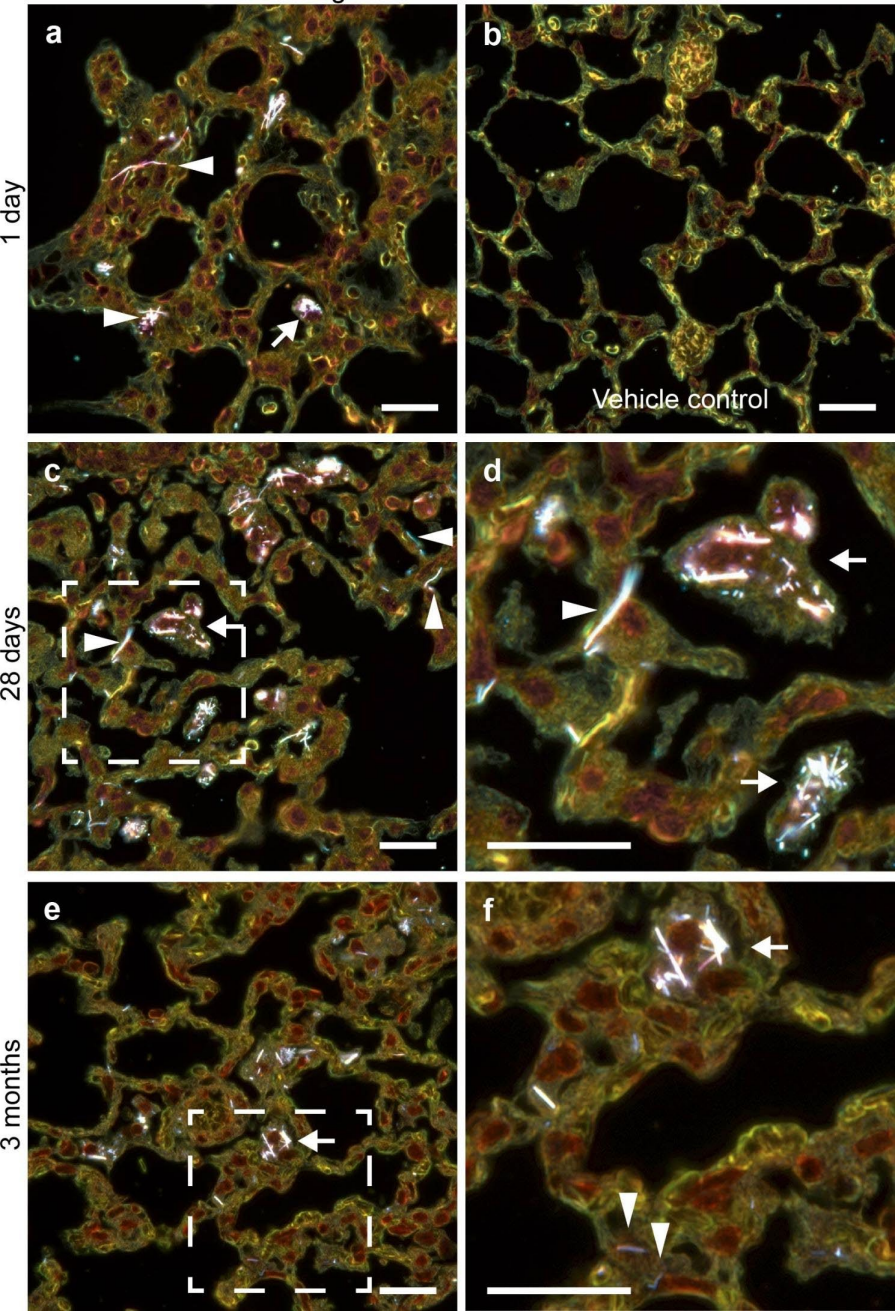
Distribution of GaP NWs in the mouse lung. Enhanced darkfield microscopy images of lung tissue 1 day **(a, b)**, 28 days **(c, d)** and 3 months **(e, f)** after intratracheal instillation. Single NWs (white) and bundles of NWs in the alveolar region were phagocytized by macrophages (arrows) or located in or at alveolar walls (arrowheads). b) vehicle control. d) and f) correspond to the marked regions in c) and e) at higher magnification. Hematoxylin and eosin stain; scale bars 20 μm

**Fig. 3:**
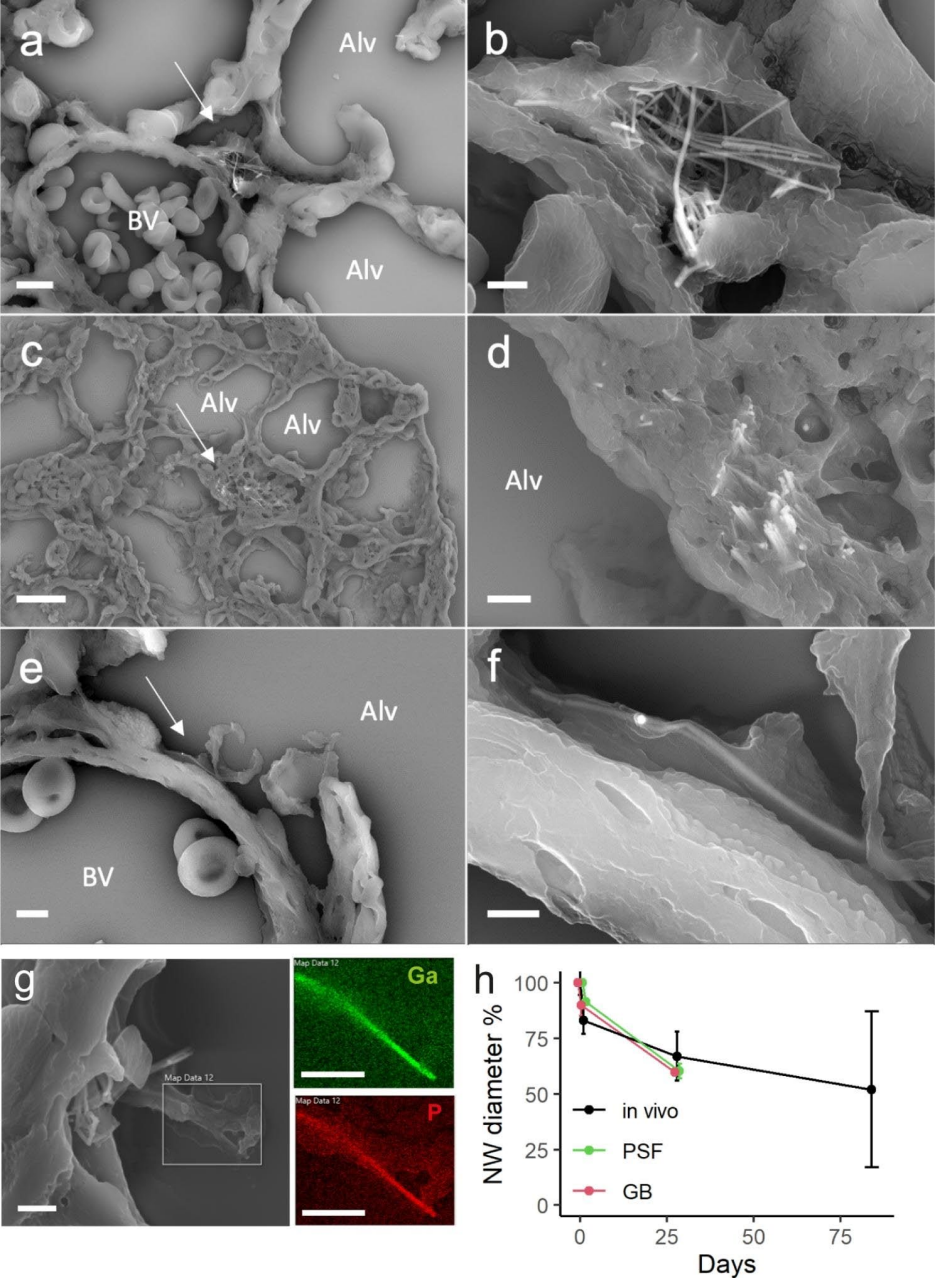
SEM images of GaP NWs embedded in lung tissue day 1 **(a, b)**, day 28 **(c, d)** and 3 months **(e, f)** post-exposure. The right panel images show the NWs indicated by arrows in the left panels at higher magnification. Alv: alveoli, BV: blood vessel. Scale bars 4 μm (a), 1 μm (b), 10 μm (c), 1 μm (d), 2 μm (e) and 0.5 μm (f). (g) Chemical identification of gallium (Ga) and phosphorus (P) in NWs in lung tissue 1 day after exposure. SEM and EDS, scale bars 1 μm. (h) NW dissolution in vivo in lung tissue and in vitro in simulated phagolysosomal fluid (PSF) and simulated lung lining fluid (Gamble’s, GB). N = 3, at least 30 NWs were measured per time point

To assess the distribution and persistence of GaP NWs in lungs, histological sections of lung tissues at 1 day, 28 days and 3 months after instillation were imaged using enhanced darkfield microscopy (Fig. 2). The GaP NWs showed intense light scattering in enhanced darkfield and were therefore easily detected in tissues. One day after instillation, NWs were mainly distributed in the alveolar region close to terminal bronchioles, either as single, straight fibers or as bundles of fibers (Fig. 2a). Many NWs were phagocytized by alveolar macrophages (Fig. 2a, arrow), and others appeared at or in alveolar walls (Fig. 2a, arrowheads). After 28 days, there were focal areas near terminal bronchioles with a high density of NWs (medium NW density shown in Fig. 2c, d). After 3 months, there were still focal areas with high density of NWs, including macrophages loaded with NWs (Fig. 2e, f), whereas some parts of the lung tissue distal from airways had no NWs or mainly single scattered NWs in or at alveolar walls, or located closely around blood vessels (Figure S3). Some NWs showed reduced light scattering after 3 months in lung (Fig. 2f, arrowheads). SEM images showed that the NWs were embedded in lung tissue, and the NWs in the lung sections were unequivocally identified by their chemical composition using energy dispersive spectroscopy (EDS) (Fig. 3). Additional darkfield and SEM images are included as supporting information (Figure S3 – S5).

### Dissolution of GaP NWs in vitro and in vivo

Dissolution of GaP NWs was estimated by measuring the decrease in NW diameter over time in vivo in lung tissue and in vitro in simulated phagolysosomal fluid and simulated lung lining fluid (Fig. 3h and Table S4). NWs grown using MOVPE from gold seed nanoparticles retain the gold nanoparticle after growth. SEM images were used to measure the decrease in NW diameter as compared to the insoluble gold nanoparticles.

The estimated GaP NW half-life due to dissolution in vivo was approximately 3 months and somewhat less in vitro. The dissolution primarily resulted in thinner fibers. The measurements of the dissolution at the 3 month time point in vivo and day 28 in vitro are more uncertain than for the earlier time points. In vivo, the gold particles were detached from the NWs 3 months post-exposure, so normalization of the NW diameter to the accompanying gold particle was not possible. In vitro, the daily adjustment of pH of the Gamble’s solution changed the morphology of some gold particles introducing an uncertainty in the determination of the gold particle diameter after 28 days. Nevertheless, the estimated half-life is consistent with the fact that many GaP NWs could be found in lung tissue 3 months after exposure. We have previously observed degraded GaP NWs in microglial cells and/or macrophages in the rat brain 6, 12 and 52 weeks after NW implantation in the striatum [19]. When coated with hafnium oxide, the NWs were found intact in the brain 1 year after implantation [20]. The difference in NW solubility was attributed to the GaP material, which as opposed to hafnium oxide, is slowly soluble in hydrogen peroxide, which is produced by immune cells [19, 55].

A 91-day study performed in rats, using intratracheal instillation of silicon NWs of 2–15 μm in lengths and 20–30 nm in diameter, resulted in transient dose-dependent lung injury and inflammation. In that study, 70% of the NWs deposited in the lung were cleared 28 days after instillation, at doses similar to the current study when normalised to body weight. We speculate that the smaller diameter (likely resulting in a larger specific surface area) and a difference in material bio-persistence resulted in faster dissolution of silicon NWs compared to the GaP NWs used in the present study. Similarly, the observed relative solubility of the GaP NWs could be assumed to reduce their long-term toxicity as compared to the insoluble MWCNT Mitsui-7. However, the partial solubility mainly resulted in decreasing the diameter of the NWs, which consequently maintained their high aspect ratio.

We have recently shown that thick and straight MWCNTs, including Mitsui-7, were dispersed in lung tissue as single fibers 1 year after pulmonary exposure in mice [38]. In contrast, short, thin, and entangled MWCNTs were encapsulated in granulomas. In the current study, GaP NWs were observed both as free single fibers and in agglomerates engulfed by macrophages at 1 day to 3 months post-exposure. This suggests that single GaP NWs can avoid uptake by macrophages or escape endosomal vesicles after uptake, similar to thick and straight MWCNTs [37]. Interestingly, our previous findings in the rat brain showed that most 2 μm long NWs were internalized by macrophages, whereas a substantial number of 5 μm long and 10 μm long NWs were not internalized by macrophages [20]. The reasons behind this could not be determined with certainty, but it would be consistent with the fiber paradigm and the hypothesis of frustrated phagocytosis [5].

### Pulmonary inflammation evaluated by cellular composition of bronchoalveolar lavage

Particle-induced lung inflammation is characterized by an influx of inflammatory cells into the lungs, which is assessed via the cellular composition of bronchoalveolar lavage (BAL) fluid. Neutrophil influx is a hallmark of an inflammatory response, whereas eosinophil influx indicates an allergic response [50]. To evaluate the inflammatory response to GaP NW exposure, inflammation was assessed in terms of the cellular composition in BAL fluid 1, 3, 28 days and 3 months post-exposure, and compared to MWCNT-induced inflammation. A single dose of carbon black (162 µg) was included as a benchmark of particle-induced pulmonary inflammation to allow comparison with previous studies [39, 41, 47, 56–65].

**Fig. 4:**
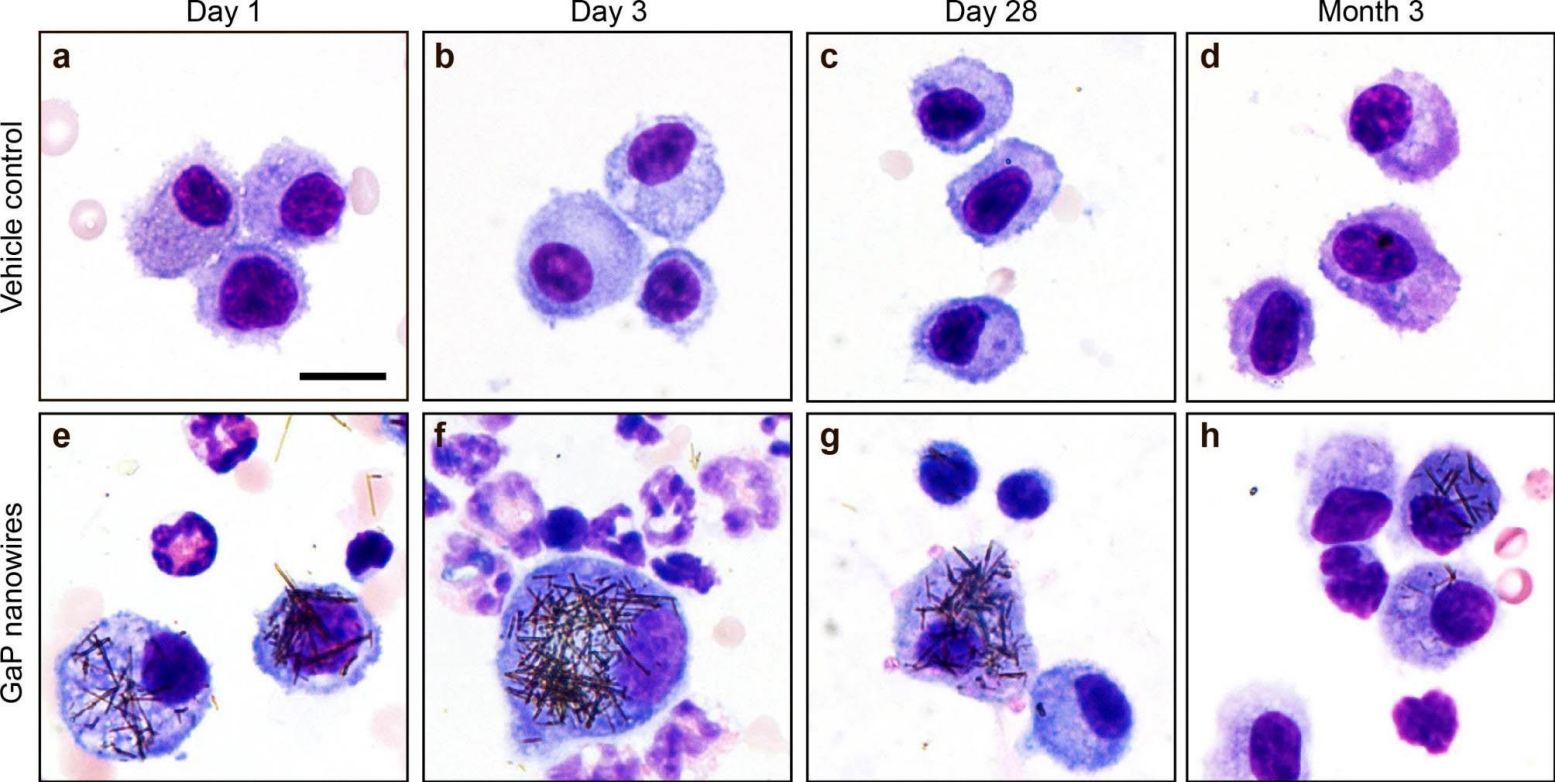
Brightfield microscopy images of macrophages in bronchoalveolar lavage 1, 3, 28 days and 3 months after intratracheal instillation of vehicle (a-d) and GaP NWs (e-h). GaP NWs appear as straight black fibers. Red blood cells, eosinophils, neutrophils and lymphocytes can be seen in the images. May-Grünewald-Giemsa stain. Scale bar 10 μm applies to all panels

GaP NWs were visible as straight black fibers in BAL macrophages at all post-exposure time points using brightfield microscopy (Fig. 4).

**Fig. 5:**
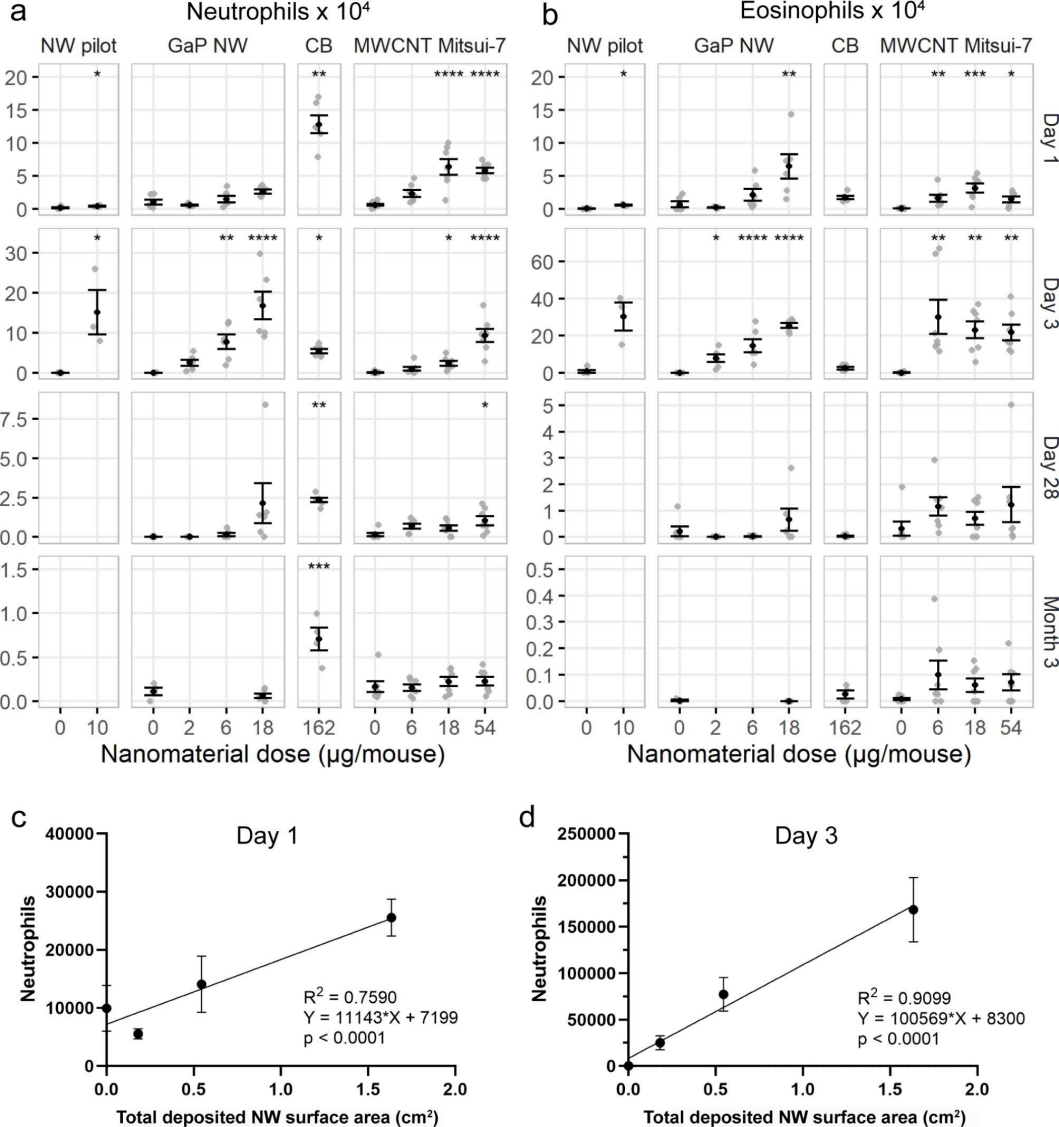
Number of neutrophils **(a)** and eosinophils **(b)** in bronchoalveolar lavage fluid 1, 3, 28 days and 3 months after exposure to GaP NWs, carbon black Printex 90 (CB) or MWCNT Mitsui-7. Dot plot with mean ± standard error of the mean. Sample size: n = 5 for 0 µg GaP NW pilot; n = 3 for 10 µg GaP NW pilot; n = 6 for 0, 6, 18 µg GaP NW and 162 µg carbon black; n = 7–9 for 0 µg MWCNT; n = 7 for 6, 18 and 54 µg MWCNT. Statistical significance compared to controls *p < 0.05, **p < 0.01, ***p < 0.001, ****p < 0.0001. **(c, d)** Linear regression analyses of the total surface area of the pulmonary deposited NWs as predictor of neutrophil influx at day 1 and day 3 post-exposure

GaP NW exposure was found to induce similar neutrophilic and eosinophilic responses as the benchmark material Mitsui-7 MWCNT, and with similar potency (Fig. 5). In general, the greatest statistically significant effects on BAL cell composition of GaP NWs and MWCNTs were observed 3 days post-exposure, as previously observed for other high aspect ratio nanomaterials [36, 37, 50, 60].

A dose-dependent, increased number of eosinophils was observed on day 1 and 3 post-exposure for GaP NWs in both the pilot study and in the main study (Fig. 5b). The time dependency and potency of the NW-induced eosinophil influx was similar to the eosinophil influx induced by Mitsui-7 MWCNTs. It cannot be ruled out that dissolution and release of ions may contribute to the observed eosinophil influx, as gradually dissolving NiO NPs induce eosinophil influx with the same time-dependency [66]. However, gallium dosed as Ga_2_O_3_ microparticles by intratracheal instillation was only mildly toxic at a 100 times higher dose level than used here [67], and phosphorus in the form of phosphate has been shown to be lethal in pigs at a corresponding phosphorus dose of approximately 1 g/kg bw [68], which is 3 orders of magnitude the highest administered dose here. Therefore, we consider it less likely that the eosinophilia induced by GaP NWs in the current study is caused by the released ions.

GaP NW exposure induced dose-dependent neutrophil influx on day 3 post-exposure considering both the pilot study and the main study (Fig. 5a). At 28 days post-exposure, the neutrophil influx was still higher than for the control group, however, it was not statistically significant. MWCNT exposure induced increased neutrophil influx at 1, 3 and 28 days post-exposure. At day 28 post-exposure, only the highest dose level of MWCNT induced neutrophil influx. The highest dose of MWCNTs was 3-fold higher than the highest dose of GaP NWs. Consequently, the potency of the MWCNTs and the NWs should be compared at the dose levels of 6 and 18 µg. Thus, the inflammatory responses of NWs and MWCNTs seemed to follow similar dose-response relationships over time.

**Fig. 6:**
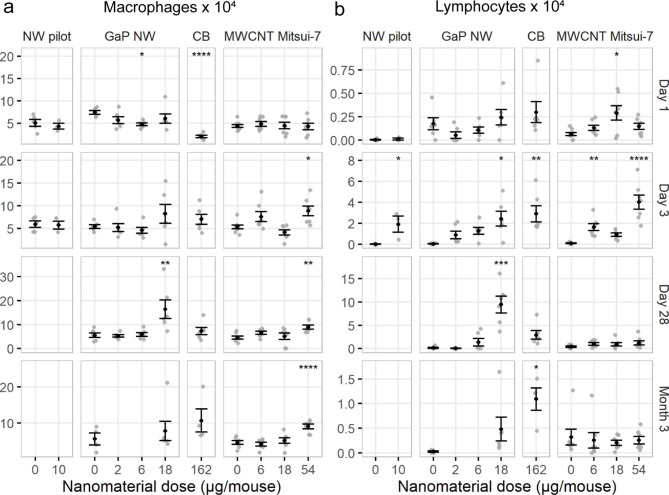
Number of macrophages **(a)** and lymphocytes **(b)** in bronchoalveolar lavage fluid 1, 3, 28 days and 3 months after exposure to GaP NWs, carbon black Printex 90 (CB) or MWCNT Mitsui-7. Dot plot with mean ± standard error of the mean. Sample sizes are listed in the legend of Fig. 5. Statistical significance compared to controls *p < 0.05, **p < 0.01, ***p < 0.001, ****p < 0.0001

Compared to vehicle controls, macrophage cell numbers were lower on day 1 post-exposure for the middle dose GaP NWs only, and higher on day 28 for the highest dose of GaP NWs (Fig. 6a). For MWCNT-exposed mice, macrophage cell numbers were higher than for vehicle controls for the highest dose level at days 3 and 28, as well as 3 months post-exposure. GaP NW exposure induced dose-dependent increasing numbers of lymphocytes 3 and 28 days post-exposure, whereas MWCNT exposure only showed an increase in the number of lymphocytes 1 day post-exposure for the middle dose and at day 3 post-exposure for the low and the high dose levels (Fig. 6b). The total number of BAL cells is included in the supporting information (Figure S6 and Table S5 and S6). A dose-dependent increase in the total number of BAL cells was observed for GaP NWs at day 3 and 28 post-exposure and for MWCNTs at 1, 3, 28 days and 3 months post-exposure, especially at the highest dose level, which is 3 fold higher than the highest GaP NW dose level. Taken together, GaP NW exposure induced similar neutrophilic and eosinophilic responses as Mitsui-7 MWCNT, with similar potency.

We have previously shown that pulmonary exposure to nanomaterials, including MWCNTs, induces inflammation that is accompanied by pulmonary and hepatic acute phase responses, which can be causally linked to the risk of coronary heart disease [69–72].

For nanomaterial-induced inflammation, the deposited surface area has been shown to predict pulmonary inflammation in terms of neutrophil influx [39, 49, 60, 73, 74]. This allows for prediction of toxicity in terms of inflammation based on the physico-chemical properties of the material. Linear regression analyses were carried out in order to assess the total surface area of the pulmonary deposited NWs as a predictor of neutrophil influx. A high correlation of total deposited NW surface area and inflammation was observed 1 day post-exposure (R^2^ = 0.759, p < 0.0001) and 3 days post-exposure (R^2^ = 0.9099, p < 0.0001) (Fig. 5c, d). This suggests that for NWs too, the total deposited surface area predicts neutrophil influx as previously reported for other high aspect ratio nanomaterials [39, 56, 60]. In addition to surface area, the fiber-shape, described by a high aspect ratio also contributes to inflammation, especially for fibers that are long enough to prevent successful phagocytosis by macrophages [5, 75]. The resulting frustrated phagocytosis is a hallmark of asbestos-induced carcinogenesis [5]. As discussed for the eosinophilic response, the observed dissolution of NWs and possible release of ions is less likely to cause tissue injury and contribute to the pulmonary inflammation as seen for the highly soluble metal oxides ZnO and CuO [57, 59, 76–78].

### Genotoxicity

**Fig. 7:**
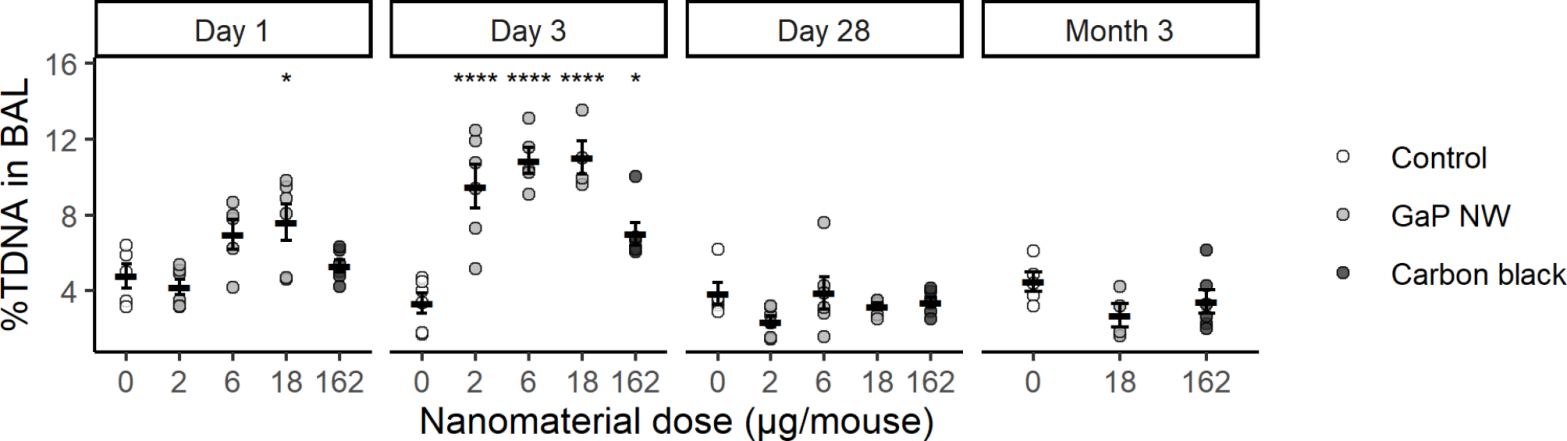
DNA strand breaks in bronchoalveolar lavage (BAL) cells 1, 3, 28 days and 3 months post-exposure. %TDNA: percent tail DNA. Dot plot with mean ± standard error of the mean. Sample size: n = 6. Statistical significance *p < 0.05, **p < 0.01, ***p < 0.001, ****p < 0.0001

DNA strand break levels were assessed in BAL cells, lung and liver tissue by the comet assay. In BAL cells, GaP NW exposure induced increased levels of DNA strand breaks at the highest dose level 1 day post-exposure and at all dose levels 3 days post-exposure (Fig. 7), but not at other time points nor in lung or liver tissues (Figure S7 and Table S7). The positive control, carbon black Printex 90 nanoparticles, also induced increased DNA strand break levels in BAL cells 3 days post-exposure. We have previously reported MWCNT-induced DNA strand breaks [36, 38, 39, 43]. The DNA strand break level correlated with increasing MWCNT diameter, which we interpreted as being a proxy for being stiff and needle-like. The specific MWCNT, Mitsui-7, has been shown to induce lung cancer in rats following inhalation exposure and has been classified as possibly carcinogenic to humans by IARC [26]. In addition, other long and straight as well as shorter and more entangled MWCNTs have been shown to induce cancer after pulmonary dosing [79–81]. Thus, the observed genotoxicity of GaP NWs is consistent with previous reports that pulmonary exposure to carbon nanotubes also induces genotoxicity.

### Histopathology of mouse lung

**Fig. 8:**
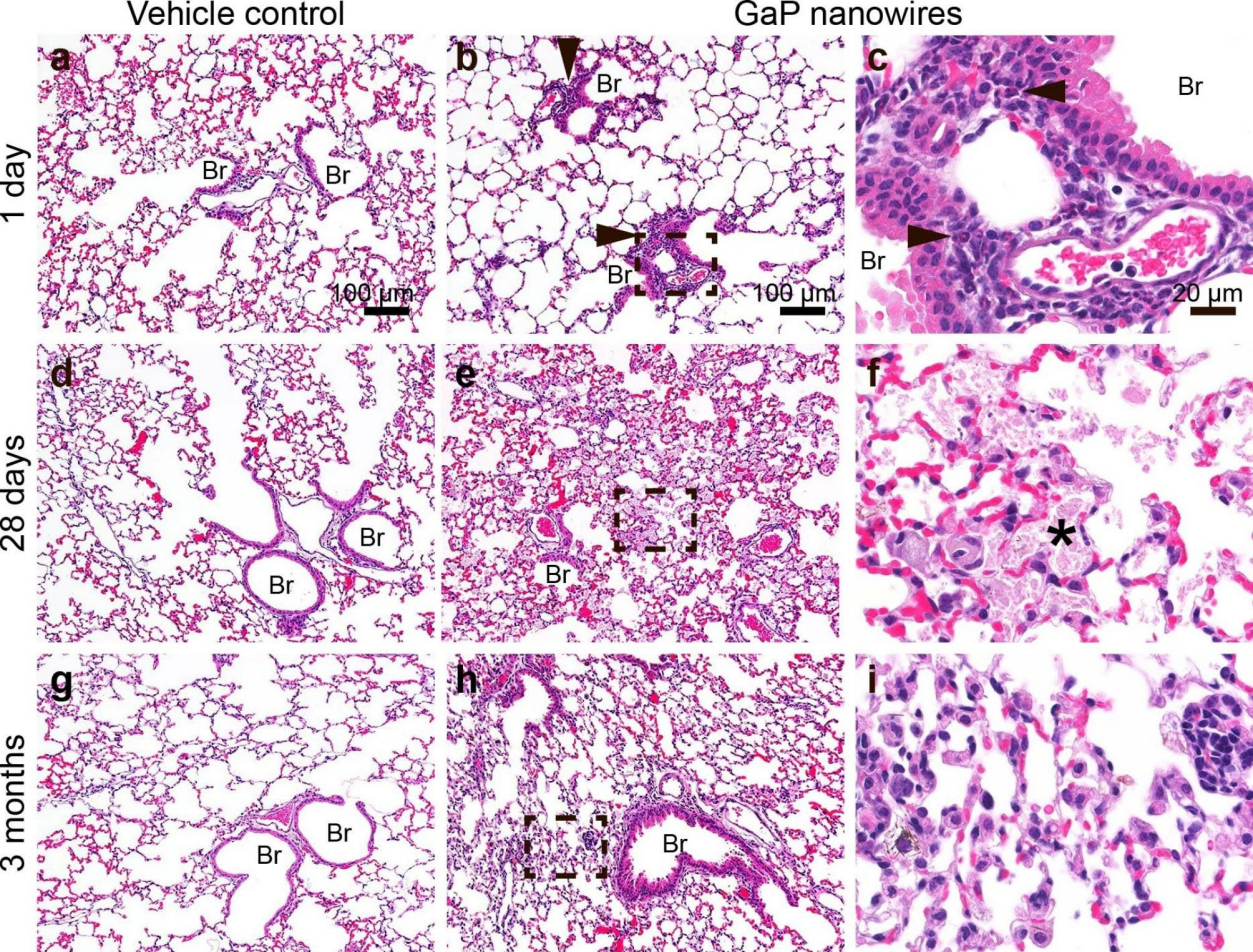
Histopathology of mouse lung 1 day, 28 days and 3 months after pulmonary exposure to 18 µg GaP NWs. Day 1: Inflammatory infiltrates with prominent eosinophils (arrowheads). Day 28: Pulmonary alveolar proteinosis (asterisk). 3 months: Histological changes receded. Brightfield microscopy of H&E stained tissue. N = 3 at 1 day and 3 months. N = 5 at day 28. Panels c), f) and i) correspond to the marked regions in b), e) and h) at higher magnification

Histopathological examination of lung sections was performed using standard brightfield microscopy (Fig. 8, incidence table in supporting information Table S8). The lung tissue from control animals 1 day, 28 days and 3 months post-exposure to vehicle appeared normal (Fig. 8a, d and g). In the lung tissue of exposed animals, NWs were seen as thin brown fibers in macrophages, though more NWs were detectable using enhanced darkfield microscopy (Figure S3). The NW exposure caused moderate inflammatory infiltrates with prominent eosinophils (Fig. 8b and c, arrowheads). This was typically seen in the peribronchial area and occasionally in the alveolar region. The observed infiltrations of inflammatory cells in lung tissue is consistent with the increased number of neutrophils and eosinophils seen in bronchoalveolar lavage. Twenty-eight days after NW instillation at the high dose, we observed diffuse macrophage activation and some inflammation in the alveolar parenchyma with few eosinophils (results not shown). Giant cells and areas with alveolar protein debris were observed in all animals exposed to the highest dose of NWs (Fig. 8f, asterisk). Three months after exposure, the changes had receded and there appeared to be fewer NWs present in the lung tissue.

In the pilot study, eosinophils were still present in lungs after 28 days and eosinophilic crystals were observed in one of two mice exposed to GaP NWs (Figure S2). In the main study, the eosinophilic response was less pronounced at day 28 than in the pilot study, and only one macrophage with eosinophilic crystals was found. Eosinophilic crystals are, in general, a rare histological observation. Therefore, although they were only observed in a few mice, it is an additional indicator of a sustained eosinophilic response. A pronounced eosinophilic response indicates an involvement of T helper 2 (Th2) cells – a Th2-type inflammation which can also be observed in an allergic response. An eosinophilic response with eosinophilic crystals and other features compatible with a Th2-type inflammation has so far been described for asbestos exposure and for two high aspect ratio nanomaterials, the MWCNTs Mitsui-7 and NM-401 [37, 50, 82–84]. Thus, GaP NW exposure appears to induce histological features similar to an eosinophilic inflammation, as previously only shown for asbestos and the MWCNTs Mitsui-7 and NM-401.

### Histopathology and GaP NW distribution in secondary organs

**Fig. 9:**
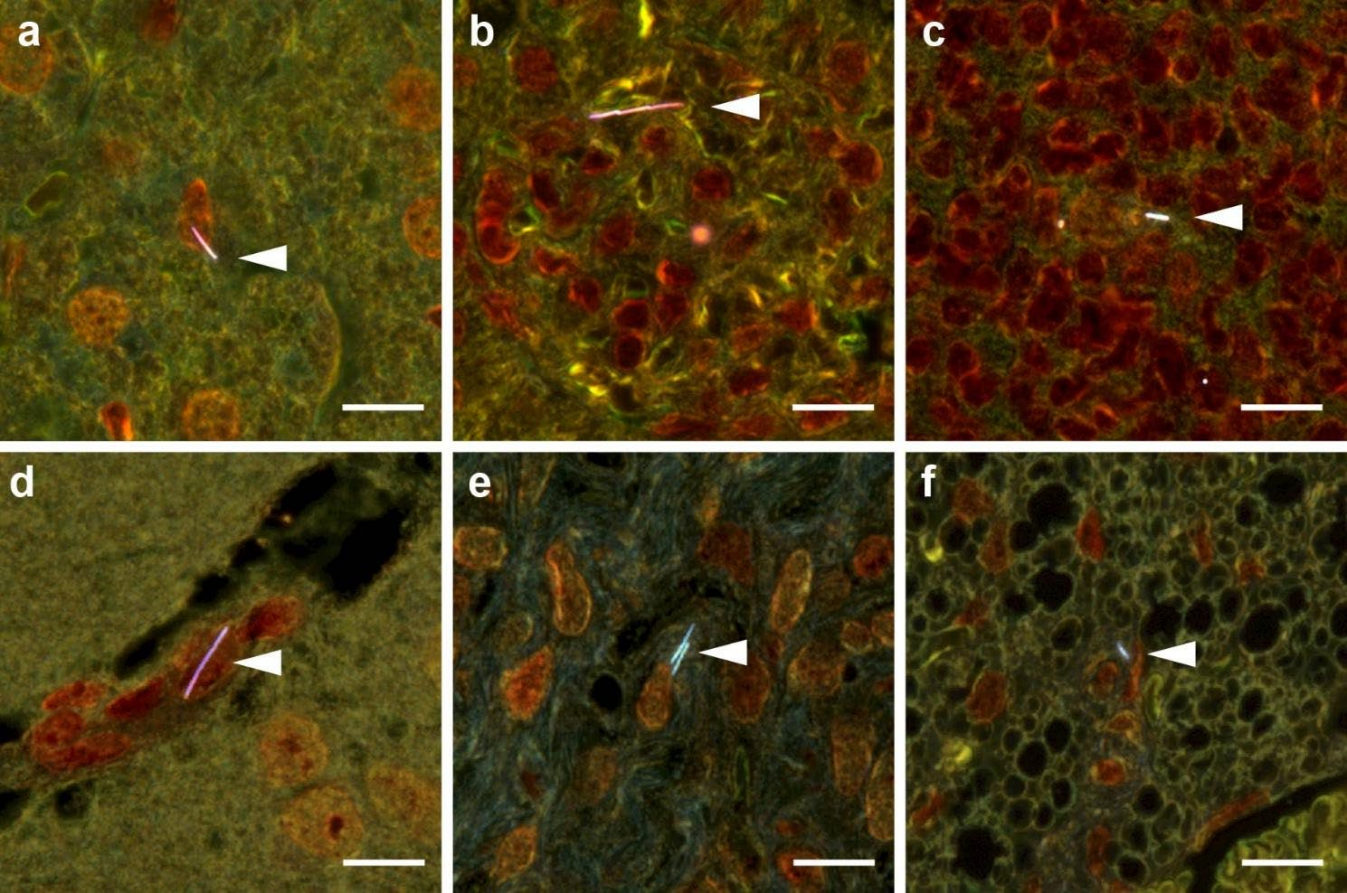
NW translocation to distant organs 28 days **(a-c)** and 3 months **(d-f)** after intratracheal instillation of GaP NWs. Enhanced darkfield microscopy of H&E stained tissue sections showing GaP NWs in **(a)** liver, **(b)** glomerulus in kidney, **(c)** white pulp in spleen, **(d)** brain vasculature, **(e)** uterus and **(f)** ventral spinal brown fat. White arrowheads indicate GaP NWs. Scale bars 10 μm

No histopathological changes that could consistently be associated with exposure were observed in liver, kidney, spleen, diaphragm, chest wall, ovaries, uterus or brain at any post-exposure time point. Possible translocation of NWs to these tissues was assessed using enhanced darkfield microscopy (Fig. 9; Table 1). Isolated NWs were found 1, 28 days and 3 months post-exposure in the liver (Fig. 9a), in glomeruli and between tubules in kidney (Fig. 9b) as well as in white pulp in spleen (Fig. 9c). At 3 months post-exposure, NWs were also found in brain vasculature (Fig. 9d) and in uterus (Fig. 9e). In samples of diaphragm and chest wall, NWs were found in between skeletal muscle cells (Figure S3) and in brown fat on the ventral side of the spinal cord (Fig. 9f). Since the NWs were found in specific sub-compartments of the organs, it points to a biological translocation and not an artefact of sample preparation. Similar observations have been reported following inhalation and pulmonary dosing of Mitsui-7 [27, 85] and a radioactively labelled MWCNT [86]. This suggests that GaP NWs, like MWCNTs, are able to translocate from lung to systemic circulation and thereby reach secondary organs.

**Table 1:** Incidence of secondary organs with NWs detected by enhanced darkfield microscopy. N = 3–5

	Exposure	Kidney	Liver	Spleen	Brain	Uterus body (cervix)	Right ovary	Left ovary	Right uterus horn	Left uterus horn	Chest wall	Dia- phragm
Day 1												
	Control	0/3	0/3	0/3								
	GaP NW	2/3	1/3	2/3								
Day 28												
	Control	0/5	0/5	0/5								
	GaP NW	5/5	5/5	5/5								
Month 3												
	Control	0/5	0/5	0/5	0/3	0/3	0/3	0/3	0/3	0/3	0/3	0/3
	GaP NW	3/3	3/3	3/3	3/3	3/3	1/3	0/2	0/3	1/2	2/3	3/3

### Comparison to the benchmark nanomaterials

Pulmonary toxicity of MWCNTs has been extensively studied, and there is evidence that many different MWCNTs are carcinogenic in animal studies [27, 79–81, 87], emphasizing the potential toxicity of all insoluble high aspect ratio nanomaterials. To the best of our knowledge, this is the first study on NW-induced toxicity following pulmonary exposure. We used pulmonary dosing by intratracheal instillation, which is suitable for hazard comparison, and have benchmarked GaP NWs against carbon nanoparticles and the MWCNT Mitsui-7. Taken together, our study shows that the GaP NWs induced pulmonary inflammation with similar potency as the MWCNT Mitsui-7 with similar physical dimensions. The inflammatory response was characterized by neutrophil and eosinophil responses. Compared to the Printex90 carbon nanoparticles, the NWs induced a stronger neutrophil influx on day 3 as previously observed for other fiber-like nanomaterials [36, 37, 60], in contrast to spherical particles where the inflammatory response declines from day 1 [65, 88–90]. GaP NWs also induced genotoxicity in BAL cells 3 days post-exposure. Similar to MWCNTs, GaP NWs were able to translocate from the lung to reach secondary organs throughout the body [91]. The GaP NWs were somewhat soluble with an estimated half-life due to dissolution in lung of approximately 3 months. Of note, the dissolution resulted in thinner NWs, so the NWs still maintained their fiber-shape and high aspect ratio. In comparison, MWCNTs are insoluble in lung in vivo, and the half-life for pulmonary clearance following inhalation exposure was estimated to be approximately one year for Baytubes MWCNTs [92]. Assuming that this slow clearance rate is representative of Mitsui-7 MWCNTs, this suggests a faster clearance of GaP NWs than Mitsui-7 MWCNTs, which would be expected to reduce the NW-induced long-term toxicity as compared to MWCNTs. We have previously estimated that exposure to 0.000 03 mg/m^3^ (30 ng/m^3^) MWCNT Mitsui-7 during a 45-year work life would lead to one excess lung cancer case per 1000 exposed [93]. However, the long-term toxicity of GaP NWs following repeated occupational exposure warrants further studies.

## Conclusion

In conclusion, pulmonary exposure to GaP NWs induced acute pulmonary inflammation with similar potency as the MWCNT Mitsui-7. GaP NW exposure also induced genotoxicity in BAL cells. However, the GaP NWs underwent dissolution in vivo, resulting in thinner NWs. The observed dissolution would be expected to reduce the long-term toxicity of the NWs. The combined effect of NW dissolution and high acute toxicity should be assessed in long-term studies. Despite the observed partial dissolution, NWs were detected in multiple secondary organs 3 months post-exposure. Based on the acute responses observed, we recommend that precautionary measures should be implemented to prevent any potential human exposure in production and post-production scenarios.

## Materials and methods

### GaP NW synthesis

Gallium phosphide (III-V semiconductor) NWs were synthesized using gold particle assisted metal-organic vapor-phase epitaxy (MOVPE) by adapting a previously reported protocol [94]. The MOVPE chamber was preconditioned before each GaP nanowire synthesis. The chamber preconditioning procedure consists of a 60 min long etch at 750 °C using HCl, followed by a 30 min GaP deposition at 630 °C, using trimethylgallium (TMGa) and Phosphine (PH3) at a molar fraction of 4.2$\times$10^−5^ and 3.8$\times$10^−3^, respectively. The preconditioning step ensures reproducible GaP NW synthesis, without contaminants.

GaP (111B) substrates were covered with 80 nm Au nanoparticles at 2 particles/µm^2^ density using aerosol deposition [95] and transferred to the MOVPE reactor (Aixtron 200/4). At 650 °C temperature and hydrogen – phosphine (H_2_/PH_3_) atmosphere the native oxide from substrates was removed and Au particles were annealed to the substrates. After 10 min, the temperature in the reactor was reduced to 440 °C and NW growth precursors, TMGa and phosphine PH_3_ at molar fractions of 8.9 × 10^− 6^ and 6.9 × 10^− 3^, respectively, were introduced into the reactor for 90 min. Additionally, HCl was introduced at a molar fraction of 3.1 × 10^− 3^ to reduce the radial growth.

### Characterization of NWs in suspension

The NW suspension for mice instillation was prepared as follows. First, the NW substrates were plasma treated (Asher – PlasmaPreen) for 30 s at 5 mbar O_2_ to make the NWs hydrophilic. NW substrates were then immediately transferred to an Eppendorf tube containing nanopure water (Nanopure Diamond UV (Barnstead) with a 0.2 mm filter (g-irradiated Barnstead D3750 hollow fiber), resistivity > 18.2 MΩ.cm at 25ºC, total organic carbon < 3.0 ppb), where NWs were removed from the substrate using ultrasound bath (1 min) and the remaining nanowires were gently scraped off from the substrate using a plastic pipette. The NW length and concentration in the suspension were determined by depositing 1 µL of NW suspension on a silicon wafer, letting the water evaporate and counting the NWs using SEM on a defined fraction of the dried drop area (1365 µm^2^). Based on the NWs counted on the SEM images, the highest GaP NW concentration used in this study was calculated to be 0.36 µg/µL. Mouse serum was subsequently added to the NW suspension to a final concentration of 2% v/v [54].

### Surface area of GaP NWs

The total amount of synthesized GaP NWs was not sufficient to make a Brunauer-Emmett-Teller analysis of the specific surface area. Instead, the specific surface area of NWs was estimated by calculating the surface area from the measured diameter and length, as well as the bulk density of GaP of 4.138 g/cm^3^. The total deposited NW surface area was estimated by multiplying the specific surface area by the NW dose.

### Carbon black printex 90 and MWCNT Mitsui-7

Carbon black (CB) Printex 90 nanoparticles were a kind gift from Evonik Degussa (Essen, Germany). Printex 90 carbon black nanoparticles have been characterized previously and have a primary diameter of 14 nm [42, 96]. MWCNTs Mitsui-7 were a kind gift from the Mitsui Company. The current batch of Mitsui-7 (also called MWNT-7 and NRCWE-006) has previously been extensively characterized [42]. The MWCNTs have a diameter of 74 ± 28 nm and average length of 5.7 μm ± 0.49 μm. Carbon black and MWCNTs were dispersed in nanopure water with 2% v/v mouse serum using probe sonication on the day of exposure as previously described [36, 54].

### Animals

Female mice C57BL/6JRj aged 7 weeks (Janvier Labs, France) were randomly allocated to the experimental groups (Table S3) (N = 6–9 mice/group for inflammation, N = 6 for genotoxicity, N = 3–5 for histology/darkfield, N = 264 mice in total). The mice were acclimatized for 1 week before the start of the experiment. The caging conditions were as previously described [64]. Briefly, all mice were housed in polypropylene cages with bedding (sawdust) and activity enrichment at controlled environmental conditions; temperature (21 ± 1 °C), humidity (50% ± 10%) and 12 h light/dark period. Mice had access to food (Altromin 1324) and tap water ad libitum. The animals were assigned to intratracheal instillation at 8 weeks of age. The average body weight on the day of instillation was 17 ± 1 g, 17 ± 1 g and 19 ± 1 g for the GaP NW, CB and MWCNT exposure groups respectively. All procedures complied with the EC Directive 86/609/EEC and Danish law regulating experiments with animals (The Danish Ministry of Justice, Animal Experiments Inspectorate, permission 2015-15-0201-00465).

### Exposure by intratracheal instillation

The intratracheal instillation was performed as previously described [97]. Briefly, mice were anesthetized with 4% Isoflurane and instilled through the trachea with vehicle (2% mouse serum in nanopure water) or vehicle containing 2, 6 and 18 µg GaP NWs, 162 µg carbon black Printex 90 or 6, 18 or 54 µg MWCNT Mitsui-7 (50 µL solution followed by 200 µL air). After instillation, the mice were weighed and transferred to the home cage until termination.

### Termination and histology

Upon termination, the mice were anesthetized by subcutaneous injection of ZRF cocktail (Zolazepam 3.29 mg/mL, Tiletamine 3.29 mg/mL, Xylazine 0.45 mg/mL, Fentanyl 2.6 µg/mL, in sterile isotone 0.9% NaCl solution, dose 0.1 mL/10 gram bodyweight). The heart blood was withdrawn. For histology the lungs were filled slowly with 4% formalin under 30 cm water column pressure in situ. A knot was made around the trachea to secure formaldehyde in the lungs and to fixate tissue in the “inflated state”. The lungs were then removed from the chest cavity, along with the liver, kidney and spleen and placed in 4% formalin for 24 h. The right caudal lung lobe of 3 high-dose histology animals were used for electron microscopy. As a supplement, the right caudal lung lobe of 3 of the 6 high-dose lavaged animals were processed for histology. Three months post-exposure, the brain, diaphragm, chest wall, uterus and ovaries were additionally sampled for histology. After fixation, the tissues were trimmed, dehydrated on a Leica ASP300S (Leica Systems, Wetzlar, Germany) and embedded in paraffin. Sections were cut at 3 μm using a Microm HM 355 S Microtome (Thermo Scientific™, East Windsor, New Jersey, USA) and stained with Hematoxylin and Eosin (H&E) or Sirius red. Tissue sections were examined by light microscopy using a Nikon Eclipse E 800 microscope. Brightfield images were acquired at 20x and 100x using an Olympus BX 43 microscope with a Nikon DS-Fi2 camera.

### NW tissue distribution using enhanced darkfield imaging

Cytoviva enhanced darkfield hyperspectral system (Auburn, AL, USA) was used to detect GaP NWs in all tissues. H&E stained histological sections were scanned at 40x in enhanced darkfield mode. The operator was blinded to the exposure group. Images were acquired at 100x magnification, using an Olympus BX 43 microscope with a Qimaging Retiga4000R camera.

### SEM and EDS imaging of NWs in lung

Lung lobes in formalin were dehydrated in ethanol (≈ 2 days in 70% ethanol, followed by 2 h in 96% ethanol and 1 h in absolute ethanol). The lobes were then placed in a 1:1 mixture of absolute ethanol and xylene for 30 min and subsequently placed in xylene for 2 h. The lobes were subsequently embedded in paraffin before being sectioned in 4 μm thick sections, deposited on glass coverslips. The paraffin was subsequently removed by dipping the sections in xylene, then 70% alcohol, followed by distilled water (1–2 min for each bath) before air drying. The samples were coated with 5 nm 80/20 Pt/Pd (Quorum, Sputter - Q150T ES) before SEM imaging (Zeiss, GeminiSEM500) using a secondary electron detector, a back scattered electron detector and an EDS detector with EDS software (Oxford Instruments) for identifying GaP and Au materials within the tissue. In EDS, a high-energy electron beam scans the sample and X-rays are generated and detected. Elements in the sample can be unequivocally identified by their characteristic X-ray energies.

### NW dissolution in vivo and in vitro

In vivo dissolution in lungs 1 day, 28 days and 3 months after instillation was estimated by dividing the nanowire diameter by the gold nanoparticle diameter measured using SEM. N = 3 animals and at least 30 NWs in total were measured for each time point.

To calculate the percentage of NW diameter that had dissolved at day 1 and 28, we exploited that each NW was still attached to its non-dissolved gold seed nanoparticle, the diameter of which is equal to the NW diameter before dissolution (see insert in Fig. 1a). Each NW diameter was divided by the diameter of its gold nanoparticle and the ratios were averaged over the NW population for each time point. This resulted in smaller uncertainties than when dividing the average NW diameter at a given time point by the average initial NW diameter, given the broad distribution of the initial NW diameters (see NW diameter distribution in Fig. 1c). After 3 months, however, the NW dissolution was such that most NWs were detached from their gold nanoparticle (Figure S5 e, f). We therefore divided the average NW diameter at 3 months by the initial NW diameter (99 ± 24 nm).

For in vitro dissolution, NW substrates were immersed in simulated phagolysosomal fluid (PSF) and low-calcium Gamble’s solution for 1 and 28 days. The simulant fluids were prepared as described previously (Table S9 and S10) [98]. The solutions were kept in Falcon tubes in a 37 °C water bath, with the pH of PSF adjusted to 4.5 and the pH of Gamble’s solution adjusted to 7.4. The pH was constantly monitored and adjusted if deviating from the set values by adding NaOH or HCl to the solutions. Note that whereas the pH of PSF was stable over time and did not require any adjustments, the pH of Gamble’s solution was drifting to basic values and required daily addition of HCl. The addition of HCl likely had an effect on the morphology of the gold nanoparticles after 28 days. The corresponding etch rate of the NWs was measured using SEM after rinsing the substrates in DI water and air-drying them. The etching rate was calculated by measuring the ratio between NW diameter and gold nanoparticle diameter for each nanowire in the SEM images. N = 3 samples and at least 30 NWs in total were measured for each time point, except for Gamble’s day 28 where only 21 NWs were measured as many NWs were detached from their gold particle.

### Bronchoalveolar lavage (BAL) preparation

BAL cell composition was determined as previously described [64]. Lungs were flushed twice with sterile 0.9% NaCl through the trachea to obtain BAL fluid. The volume used for each flush was 1 mL 0.9% NaCl/25 g mouse weight and varied from 0.7 to 0.9 mL. The BAL fluid was stored on ice until centrifugation at 400 x g for 10 min at 4 °C. Acellular BAL fluid was recovered and stored at − 80 °C. The BAL cells were re-suspended in 100 µL medium (HAM F-12 with 1% penicillin/streptomycin and 10% fetal bovine serum). The total number of living and dead cells in BAL was determined using a NucleoCounter NC-200TM (Chemometec, Denmark) following the manufacturer’s protocol. The rest of cell re-suspension was used to determine the BAL cell composition. The cell suspension was centrifuged at 55 x g for 4 min in Cytofuge 2 (StatSpin, TRIOLAB, Brøndby, Denmark) onto a microscope glass slide and then fixed for 5 min in 96% ethanol. All slides were stained with May-Grünwald Giemsa, randomized and blinded, before counting 200 cells/sample using light microscopy at 100 x magnification.

### Genotoxicity by comet assay

Levels of DNA strand breaks were assessed in BAL cells, and lung and liver tissue as tail percent DNA, measured by comet assay using the IMSTAR Pathfinder system as previously described [99]. Negative and positive controls included on all slides were A549 cells, exposed to 0 and 45 µM H_2_O_2_, respectively. These were included to monitor day-to-day variation and efficacy of each individual electrophoresis.

### Statistical analyses

The data sets of BAL cells and DNA strand breaks were analyzed using the software package Graph Pad Prism 8.1.2. (Graph Pad Software Inc., La Jolla, CA, USA). All data are expressed as mean ± standard error of the mean. Data were tested for normality using the Shapiro-Wilks test and for variance homogeneity using the Bartlett’s test. The data were analyzed by ordinary one-way ANOVA followed by Dunnett’s multiple comparisons test as post hoc to test the differences between the test groups. The pilot BAL data were analyzed by the nonparametric Mann-Whitney test for differences between the control and the exposed group. P-value ≤ 0.05 was considered significant. The correlation between neutrophil influx and deposited NW surface area was analyzed using linear regression in Graph Pad Prism 8.1.2. (Graph Pad Software Inc., La Jolla, CA, USA).

### Electronic supplementary material

Below is the link to the electronic supplementary material.

Supplementary Material 1: Experimental details and results of the pilot study and 3-months study. GaP NW synthesis and characterization in pilot study. Table S1: Pilot study design. Figure S1: Characterization of GaP NWs in the pilot study. Table S2: (Pilot study) Cellular composition of bronchoalveolar lavage 1 and 3 days after exposure to GaP NWs. Figure S2: (Pilot study) Histopathology of mouse lung 1 and 28 days after pulmonary exposure to GaP NWs. Table S3: 3-month study design. Figure S3: Additional darkfield of GaP NWs in tissues. Figure S4: Chemical identification by EDS of GaP NWs in lung tissue 1 day after exposure. Figure S5: SEM images of GaP NWs in lung tissue day 1 and 28 and 3 months post-exposure. Table S4: Diameter of gold nanoparticles and nanowires in vivo and in vitro. Figure S6, Table S5 and S6: Cellular composition of bronchoalveolar lavage 1, 3, 28 days and 3 months after exposure to GaP NWs, carbon black or MWCNT Mitsui-7. Figure S7 and Table S7: Genotoxicity in BAL cells, lung and liver tissue in 3-month study. Table S8. Mouse lung histopathology 1, 28 days and 3 months after intratracheal instillation of GaP NWs, incidence table. Table S9: Composition of phagolysosomal simulant fluid (PSF). Table S10: Composition of low-calcium Gamble’s solution.

## Data Availability

All data generated or analysed during this study are included in this published article and the supporting information file.
