# Supplementary material for: Pulmonary toxicity and translocation of gallium phosphide nanowires to secondary organs following pulmonary exposure in mice
Source: J Nanobiotechnology. 2023 Sep 7;21:322. doi: 10.1186/s12951-023-02049-0 (PMC10483739; doi:10.1186/s12951-023-02049-0)
Supplement: Supplementary file 1 — Supplementary Material 1: Experimental details and results of the pilot study and 3-months study. GaP NW synthesis and characterization in pilot study. Table S1: Pilot study design. Figure S1: Characterization of GaP NWs in the pilot study. Table S2: (Pilot study) Cellular composition of bronchoalveolar lavage 1 and 3 days after exposure to GaP NWs. Figure S2: (Pilot study) Histopathology of mouse lung 1 and 28 days after pulmonary exposure to GaP NWs. Table S3: 3-month study design. Figure S3: Additional darkfield of GaP NWs in tissues. Figure S4: Chemical identification by EDS of GaP NWs in lung tissue 1 day after exposure. Figure S5: SEM images of GaP NWs in lung tissue day 1 and 28 and 3 months post-exposure. Table S4: Diameter of gold nanoparticles and nanowires in vivo and in vitro. Figure S6, Table S5 and S6: Cellular composition of bronchoalveolar lavage 1, 3, 28 days and 3 months after exposure to GaP NWs, carbon black or MWCNT Mitsui-7. Figure S7 and Table S7: Genotoxicity in BAL cells, lung and liver tissue in 3-month study. Table S8. Mouse lung histopathology 1, 28 days and 3 months after intratracheal instillation of GaP NWs, incidence table. Table S9: Composition of phagolysosomal simulant fluid (PSF). Table S10: Composition of low-calcium Gamble’s solution. [file 12951_2023_2049_MOESM1_ESM.docx]

# Pilot study

**Table S1**. Pilot study design. N_total_ = 26 mice.

|  |  | Dose  (µg/mouse) | Number of mice per group | | |
| --- | --- | --- | --- | --- | --- |
|  |  |  | day 1 | day 3 | day 28 |
| BAL cell composition | GaP NWs | 10 | 3 | 3 | 0 |
|  | Vehicle control | 0 | 5 | 5 | 0 |
| Histology | GaP NWs | 10 | 2 | 0 | 2 |
|  | Vehicle control | 0 | 3 | 0 | 3 |

## Animals in pilot study

Female mice C57BL/6BomTac aged 7 weeks were obtained from Taconic Europe (Ejby, Denmark). The animals were exposed by intratracheal instillation at 8 weeks of age. The average weight of the mice on the day of instillation was 20±1g. N = 26 mice in total for the NW pilot study. All procedures complied with the EC Directive 86/609/EEC and Danish law regulating experiments with animals (The Danish Ministry of Justice, Animal Experiments Inspectorate, permission 2015-15-0201-00465).

## GaP NW synthesis and characterization in pilot study

NWs for the pilot study were synthesized using MOVPE as described in the main manuscript with the exception that shorter growth times where used, resulting in quantifiable nanowire lengths immediately after growth, when nanowires are still on their substrate (Fig S1a and S1d). A NW suspension for mice instillation was prepared as follows. First, the NW substrates were plasma treated (Asher – PlasmaPreen) for 30 seconds at 5 mbar of O_2_ to make the NWs hydrophilic. NW substrates were then immediately transferred to a microtube containing 600 µL of nanopure water (Nanopure Diamond UV (Barnstead) with a 0.2 mm filter (g-irradiated Barnstead D3750 hollow fiber), resistivity >18.2 MΩ.cm at 25ºC, total organic carbon < 3.0 ppb), where NWs were gently scraped off from the substrate using a plastic pipette. The NW length and concentration in the suspension were determined by depositing 0.2 µL of NW suspension on a SiO_2_ wafer, letting the water evaporate and counting the NWs using SEM (Fig S1b). Based on the NW numbers obtained on the SEM images, we have calculated the GaP NW concentration to be 0.2 µg/µL. Mouse serum was subsequently added to the NW suspension to a final concentration of 2% v/v. The dimensions of the synthesized GaP NWs were characterized before and after removal from the substrate. The average diameter of the NWs was 78 ± 10 nm (Fig. S1 c). The average length of the NWs as grown was 13.8 µm ± 2.8 µm (Fig. S1 d) and 1.8 µm ± 3.2 µm after removal from the substrate and suspension in water (Fig. S1 e).


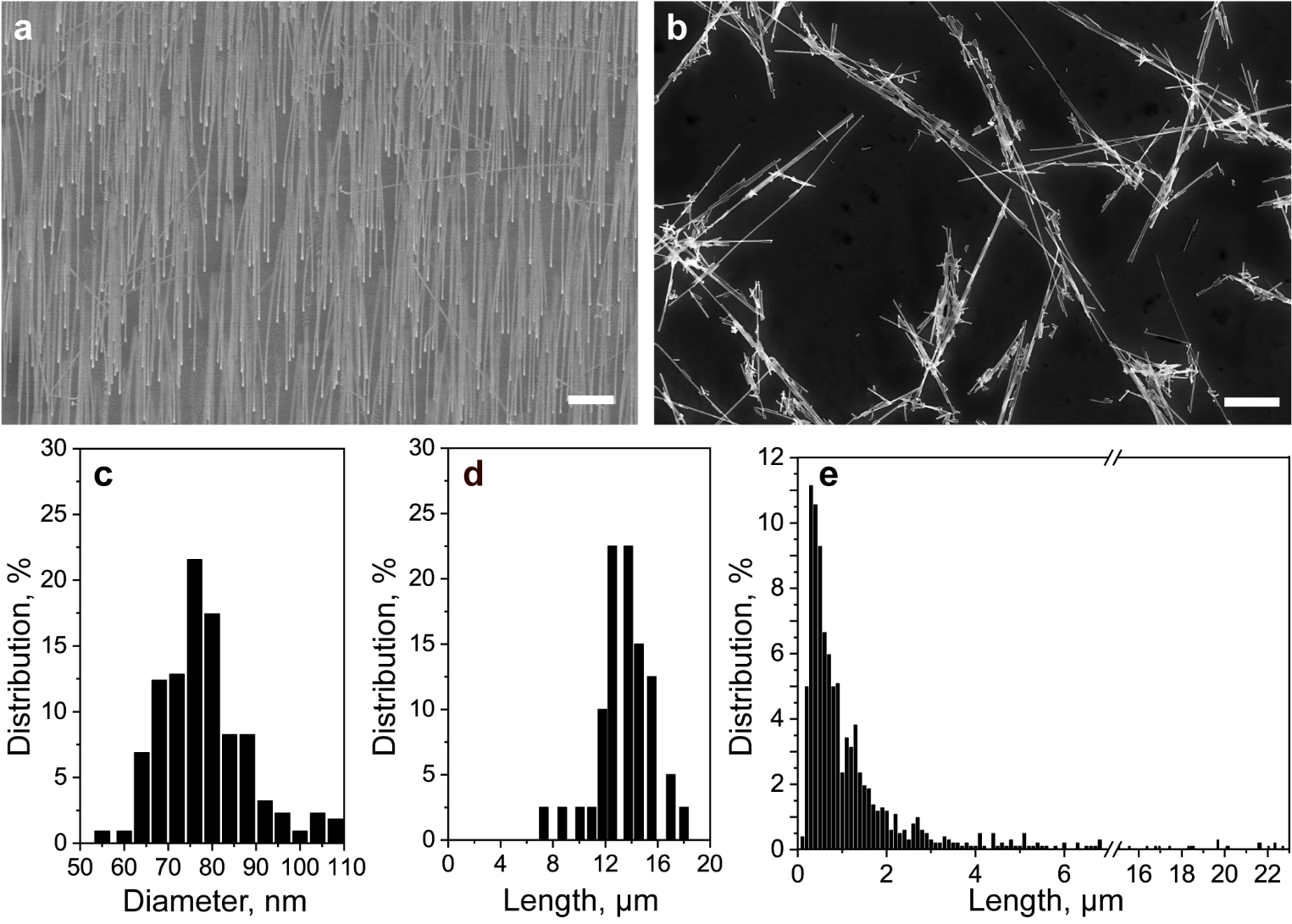


**Figure S1**. Characterization of GaP NWs in the pilot study as synthesized and after suspension. a) SEM image showing GaP NWs after MOVPE growth on the substrate. Stage tilt 30 °, scale bar 2 µm. b) SEM image showing GaP NWs after suspension. Stage tilt 0 °, scale bar 3 µm. c) GaP NW diameter distribution. d) GaP NW length distribution as synthesized. e) GaP NW length distribution in suspension.

## Cellular composition of bronchoalveolar lavage 1 and 3 days after exposure to GaP NWs in pilot study

**Table S2**. Pilot study. Cellular composition of bronchoalveolar lavage 1 and 3 days after exposure to GaP NWs (Mean +/- SEM).

|  |  | **Vehicle control** | | | | | |  | **GaP NW** | | | | | |
| --- | --- | --- | --- | --- | --- | --- | --- | --- | --- | --- | --- | --- | --- | --- |
|  |  | 0 µg | | | | | |  | 10 µg | | | | | |
|  |  | Cell count (x10^3^) | | |  | % |  |  | Cell count (x10^3^) | | |  | % |  |
| **Day 1** | |  |  |  |  |  |  |  |  |  |  |  |  |  |
|  | **Total BAL cells***^a^* | 56.9 | ± | 7.7 | 100 |  |  |  | 56.1 | ± | 7.2 | 100 |  |  |
|  | **Macrophages** | 50.9 | ± | 7.5 | 88.6 | ± | 2.4 |  | 43.5 | ± | 6.7 | 77.3 | ± | 4.1 |
|  | **Lymphocytes** | 0.03 | ± | 0.03 | 0.1 | ± | 0.1 |  | 0.1 | ± | 0.1 | 0.2 | ± | 0.2 |
|  | **Neutrophils** | 1.3 | ± | 0.9 | 1.8 | ± | 1.1 |  | 3.4 | ± | 0.8 | 6.3 | ± | 1.5 |
|  | **Eosinophils** | 0.05 | ± | 0.05 | 0.1 | ± | 0.1 |  | 5.4 | ± | 1.0 | 10.0 | ± | 2.3 |
| **Day 3** | |  |  |  |  |  |  |  |  |  |  |  |  |  |
|  | **Total BAL cells***^a^* | 71.9 | ± | 5.4 | 100 |  |  |  | 536.0 | ± | 133.3 | 100 |  |  |
|  | **Macrophages** | 59.0 | ± | 7.1 | 82.5 | ± | 8.1 |  | 57.2 | ± | 8.8 | 11.5 | ± | 1.8 |
|  | **Lymphocytes** | 0.1 | ± | 0.1 | 0.2 | ± | 0.1 |  | 19.1 | ± | 7.6 | 3.2 | ± | 0.8 |
|  | **Neutrophils** | 0.1 | ± | 0.1 | 0.1 | ± | 0.1 |  | 151.6 | ± | 55.3 | 27.8 | ± | 4.9 |
|  | **Eosinophils** | 8.2 | ± | 7.7 | 10.1 | ± | 9.4 |  | 302.1 | ± | 76.5 | 56.5 | ± | 5.0 |

*^a^*Total BAL cell count includes epithelial cells (not shown). Statistics not shown. Group size: n=5 for vehicle controls, n=3 for GaP NW.


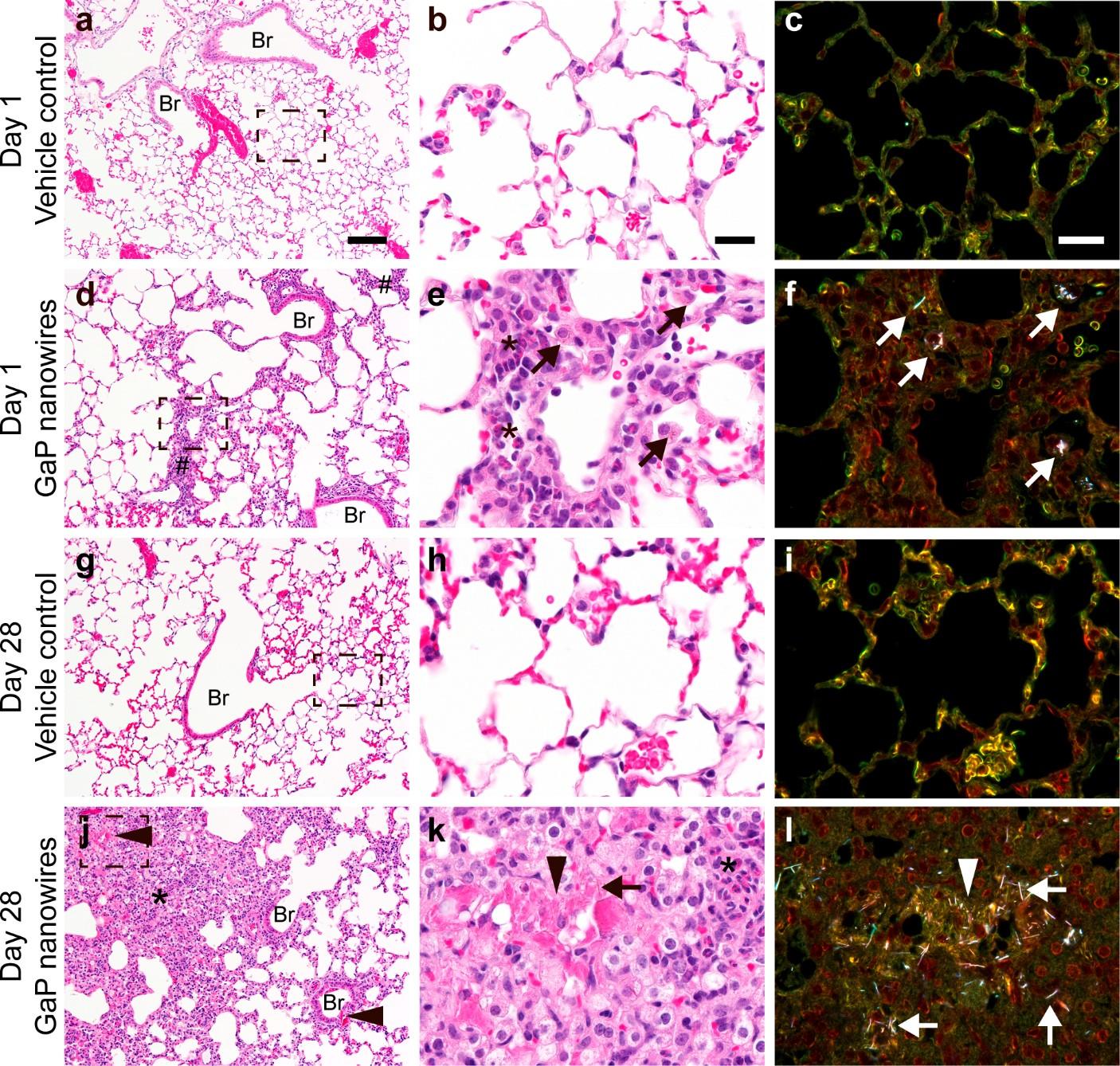


**Figure S2**. Pilot study. Histopathology of mouse lung 1 and 28 days after pulmonary exposure to GaP NWs. *infiltrate with eosinophils and other inflammatory cells, #interstitial thickening, arrow heads: eosinophilic crystals, arrows: GaP NWs, Br: terminal bronchiole. Brightfield and enhanced darkfield microscopy of H&E stained tissue; n = 3 per day for vehicle control, n = 2 per day for GaP NWs. Scale bar 100 µm applies to a, d, g, i. Scale bar 20 µm applies to b, e, h, k and panels c, f, i, l. Panels b, e, h, k correspond to the rectangular regions in panels a, d, g, i at higher magnification.

# 3-month study

**Table S3**. 3-month study design. Number of mice per group and endpoint. N_total_= 264 mice.

| Exposure | Day | Dose | Inflammation & genotoxicity | Histopathology and biodistribution* |
| --- | --- | --- | --- | --- |
| GaP NW | 1 | 0/2/6/18µg | 6 | 3 (+3 high dose) |
|  | 3 | 0/2/6/18µg | 6 |  |
|  | 28 | 0/2/6/18µg | 6 | 5 (+3 high dose) |
|  | 85 | 0/18µg | 6 | 5/3 (+3 high dose) |
|  |  |  | Total 124 | |
| Carbon black | 1 | 162µg | 6 |  |
|  | 3 | 162µg | 6 |  |
|  | 28 | 162µg | 6 |  |
|  | 87 | 162µg | 6 |  |
|  |  |  | Total 24 | |
|  |  |  | **Inflammation** |  |
| MWCNT | 1 | 0/6/18/54µg | 9/7/7/7 |  |
|  | 3 | 0/6/18/54µg | 9/7/7/7 |  |
|  | 28 | 0/6/18/54µg | 7 |  |
|  | 90 | 0/6/18/54µg | 7 |  |
|  |  |  | Total 116 | |

Note: for logistic reasons, 3-month animals were taken down on day 85, 87 and 90 post-exposure. *The right caudal lung lobe of three high dose histology animals were used for EM analysis. As supplement, the right caudal lung lobe of three of the 6 lavaged animals were processed for histology.

## Additional darkfield of GaP NWs in tissues


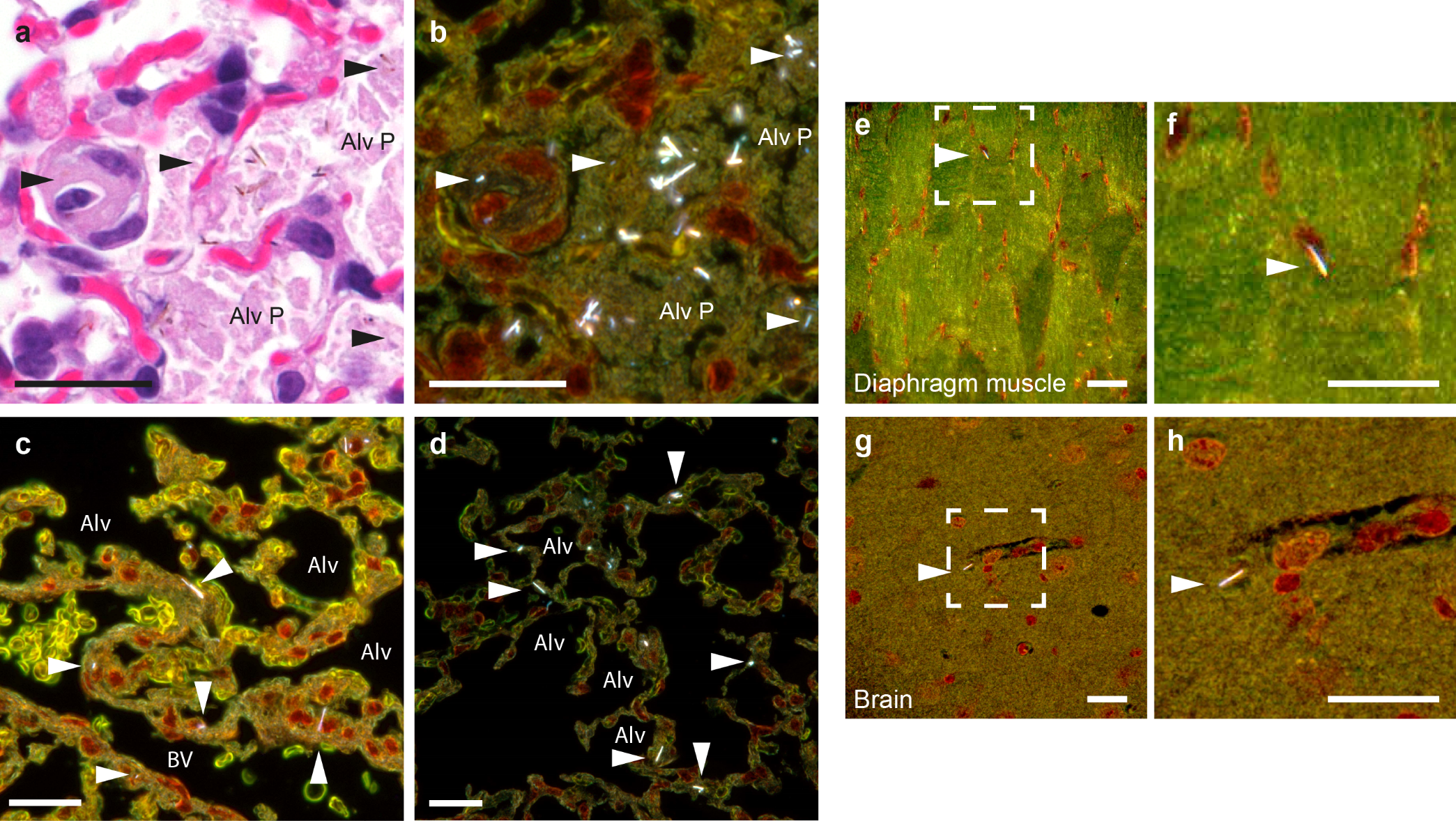


**Figure S3. GaP NWs in mouse lung (a-d), diaphragm muscle (e, f) and brain vasculature (g, h)**. **(a, b)** GaP NWs in alveolar protein debris (Alv P) 28 days post-exposure. In brightfield, some NWs were visible as thin brown fibers (a), though more NWs were detectable using enhanced darkfield microscopy (b). Arrowheads points to some of the NWs detectable in darkfield and not in brightfield. **(c, d)** 3 months after exposure, parts of the lung tissue distal from airways had no NWs or mainly single scattered NWs (arrowheads) in or at alveolar walls (d) or blood vessels walls (c). **(e, f)** NW in diaphragm muscle 3 months post-exposure. **(g, h)** NW in brain vasculature 3 months post-exposure. Brightfield (a) and enhanced darkfield (b-h). Scale bars 20 µm.

## SEM and EDS of NWs in lung in 3-month study


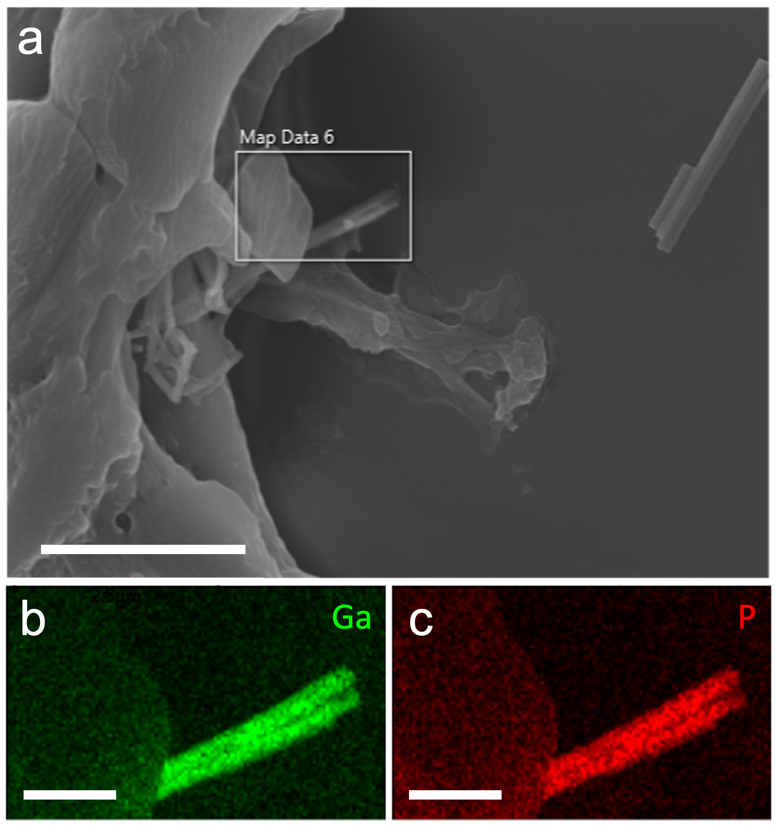


**Figure S4**. Chemical identification of GaP NWs in lung tissue 1 day after exposure. EDS was used to map the distribution of gallium (Ga) and phosphorous (P), with an incident electron energy of 10 keV. a) SEM image, secondary electron detector, scale bar 2.5 µm. b, c) scanning of the area located within the frame shown in panel (a) using the EDS detector. The nanowires are clearly distinguishable in the lung tissue by their high concentration of Ga and P. Scale bars 500 nm.


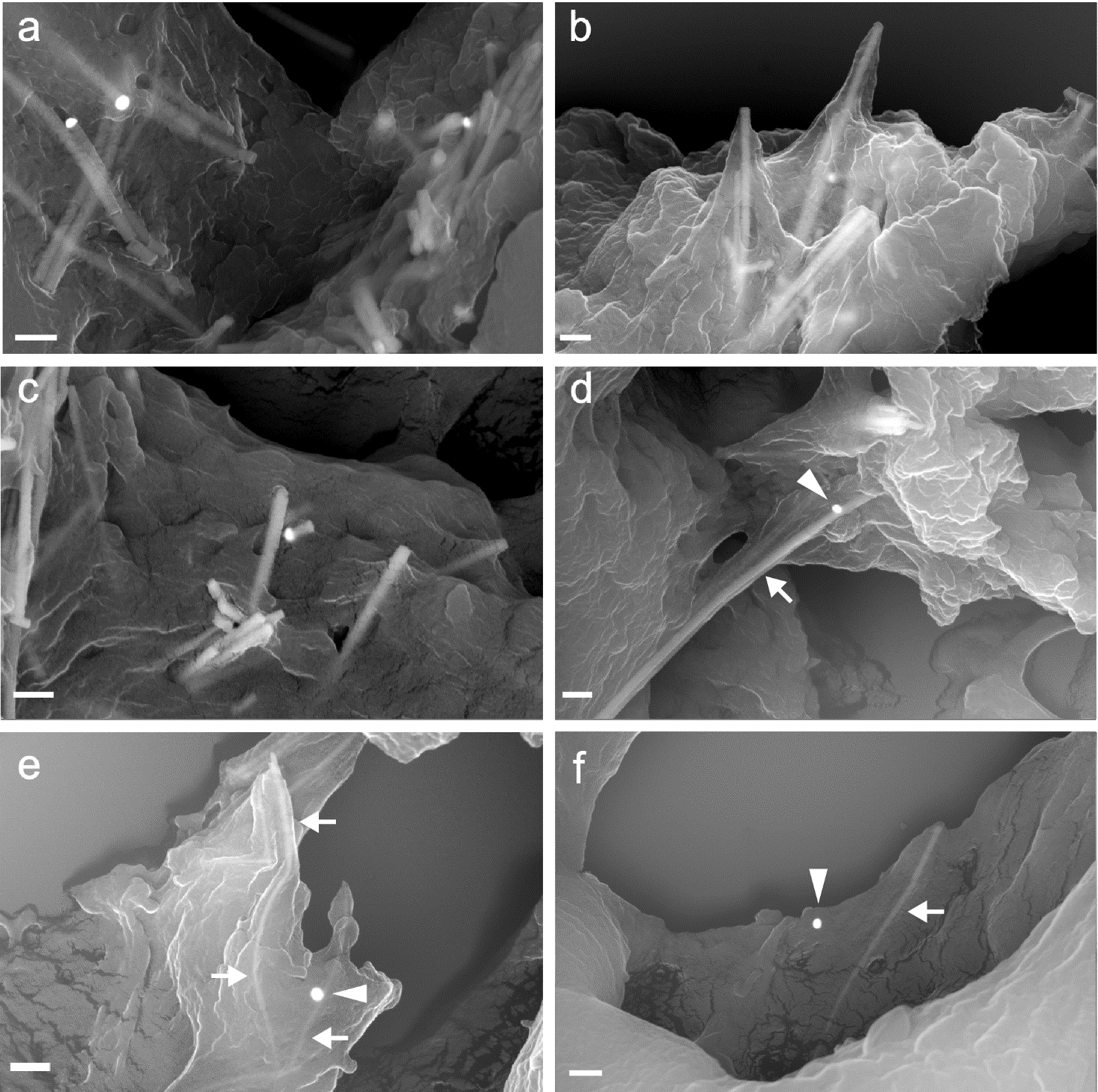


**Figure S5**. SEM images of GaP NWs in lung tissue day 1 (a, b), day 28 (c, d) and 3 months (e, f) post-exposure. Three months after exposure, many NWs (arrows) are thinner and detached from their gold particle (arrowhead) due to *in vivo* dissolution of GaP. Back scattered electron detector, scale bars 300 nm.

## **Table S4. Diameter of gold nanoparticles and NWs in lung tissue, in simulated phagolysosomal fluid (PSF) and simulated lung lining fluid (Gamble’s, GB) measured by SEM**.

Mean ± standard deviation. N = 3 and at least 30 NWs and 30 gold nanoparticles were measured for each time point.

|  | **Day 1** | **Day 28** | **Month 3** |
| --- | --- | --- | --- |
| **Average Au nanoparticle diameter in lung (nm)** | 93 ± 14 | 93 ± 14 | 85 ±2 4 |
| **Average NW diameter in lung (nm)** | 77 ± 13 | 62 ± 16 | 52 ± 22 |
| **% NW diameter in lung** | 83 ± 6 | 67 ± 11 | 52 ± 35 |
| **% NW diameter in Gamble’s** | 90 ± 5 | 60 ± 2 | ND |
| **% NW diameter in PSF** | 91 ± 0.2 | 60 ± 3 | ND |

## Cellular composition of bronchoalveolar lavage in 3-month study


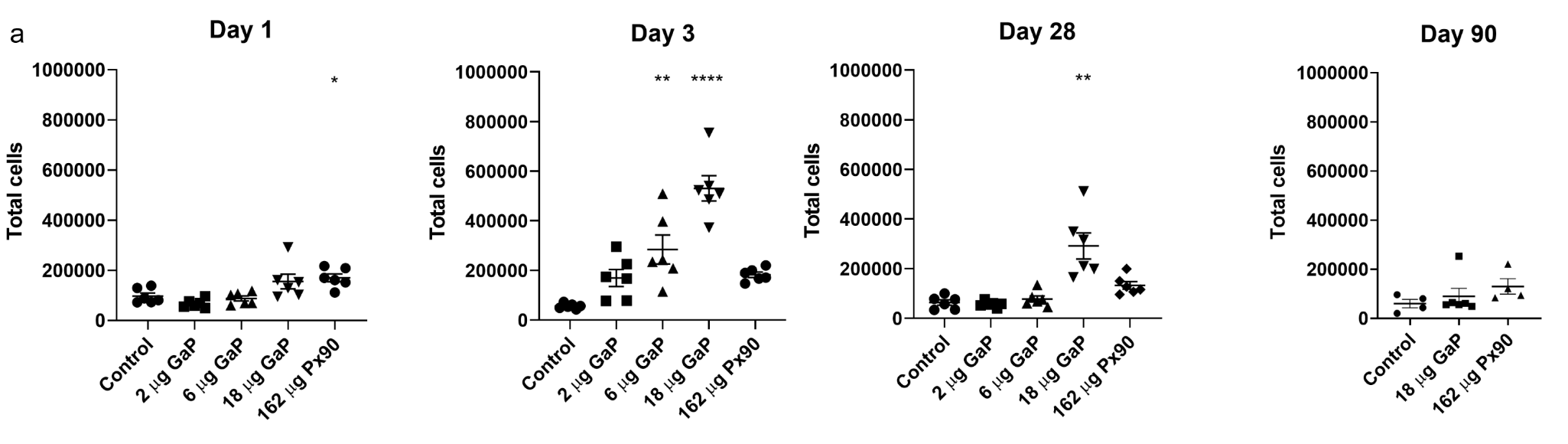


**Figure S6**. Total cell count of bronchoalveolar lavage 1, 3, 28 days and 3 months after exposure to GaP NWs (GaP) or carbon black Printex 90 (Px90). Dot plot with mean ± standard error of the mean. Sample size: n = 6. Statistical significance *p<0.05, **p<0.01, ***p<0.001, ****p<0.0001 compared to control.

**Table S5**. Cellular composition of bronchoalveolar lavage 1, 3, 28 days and 3 months after exposure to GaP NWs or carbon black (CB) Printex 90

|  |  | **Vehicle control** | | | | | |  | **GaP NW** | | | | | | | | | | | | | | | | | | | |  | **CB** | | | | | |
| --- | --- | --- | --- | --- | --- | --- | --- | --- | --- | --- | --- | --- | --- | --- | --- | --- | --- | --- | --- | --- | --- | --- | --- | --- | --- | --- | --- | --- | --- | --- | --- | --- | --- | --- | --- |
|  |  | 0 µg | | | | | |  | 2 µg | | | | | |  | 6 µg | | | | | |  | 18 µg | | | | | |  | 162 µg | | | | | |
|  |  | Cell count (x10^3^) | | |  | % |  |  | Cell count (x10^3^) | | |  | % |  |  | Cell count (x10^3^) | | |  | % |  |  | Cell count (x10^3^) | | |  | % |  |  | Cell count (x10^3^) | | |  | % |  |
| **Day 1** | |  |  |  |  |  |  |  |  |  |  |  |  |  |  |  |  |  |  |  |  |  |  |  |  |  |  |  |  |  |  |  |  |  |  |
|  | **Total BAL cells** | 97.3 | ± | 12.1 | 100 |  |  |  | 67.9 | ± | 7.0 | 100 |  |  |  | 87.8 | ± | 9.8 | 100 |  |  |  | 155.5 | ± | 29.3 | 100 |  |  |  | 170.2 | ± | 15.8 | 100 |  |  |
|  | **Macrophages** | 74.3 | ± | 3.9 | 79.6 | ± | 5.3 |  | 57.2 | ± | 7.5 | 83.2 | ± | 2.4 |  | 48.1 | ± | 3.1 | 58.7 | ± | 8.0 |  | 60.7 | ± | 10.3 | 41.1 | ± | 4.9 |  | 20.3 | ± | 2.6 | 12.2 | ± | 1.6 |
|  | **Lymphocytes** | 1.7 | ± | 0.6 | 1.7 | ± | 0.5 |  | 0.5 | ± | 0.3 | 0.7 | ± | 0.5 |  | 1.0 | ± | 0.3 | 1.2 | ± | 0.3 |  | 2.4 | ± | 0.8 | 1.7 | ± | 0.6 |  | 3.0 | ± | 1.1 | 1.8 | ± | 0.7 |
|  | **Neutrophils** | 9.9 | ± | 4.0 | 8.8 | ± | 2.7 |  | 5.5 | ± | 0.9 | 9.1 | ± | 2.1 |  | 14.1 | ± | 4.9 | 15.1 | ± | 4.3 |  | 25.6 | ± | 3.2 | 17.5 | ± | 1.8 |  | 127.8 | ± | 13.5 | 74.7 | ± | 1.9 |
|  | **Eosinophils** | 6.5 | ± | 4.2 | 4.8 | ± | 3.1 |  | 1.7 | ± | 0.4 | 2.9 | ± | 0.8 |  | 21.0 | ± | 8.8 | 20.8 | ± | 7.6 |  | 64.2 | ± | 18.6 | 37.6 | ± | 5.7 |  | 168.0 | ± | 2.5 | 9.8 | ± | 0.9 |
|  | **Epithelial** | 4.8 | ± | 1.0 | 5.1 | ± | 1.1 |  | 2.9 | ± | 0.8 | 4.1 | ± | 0.9 |  | 3.5 | ± | 1.1 | 4.2 | ± | 1.4 |  | 2.7 | ± | 0.9 | 2.1 | ± | 0.9 |  | 2.3 | ± | 0.5 | 1.5 | ± | 0.5 |
| **Day 3** | |  |  |  |  |  |  |  |  |  |  |  |  |  |  |  |  |  |  |  |  |  |  |  |  |  |  |  |  |  |  |  |  |  |  |
|  | **Total BAL cells** | 56.3 | ± | 4.3 | 100 |  |  |  | 169.6 | ± | 34.6 |  |  |  |  | 284.5 | ± | 58.3 | 100 |  |  |  | 530.7 | ± | 50.9 | 100 |  |  |  | 182.5 | ± | 10.6 | 100 |  |  |
|  | **Macrophages** | 53.8 | ± | 4.0 | 95.6 | ± | 0.6 |  | 51.8 | ± | 8.9 | 35.1 | ± | 6.7 |  | 45.7 | ± | 6.3 | 19.6 | ± | 5.1 |  | 82.0 | ± | 20.5 | 14.6 | ± | 3.0 |  | 69.9 | ± | 10.9 | 37.7 | ± | 4.4 |
|  | **Lymphocytes** | 0.5 | ± | 0.2 | 0.8 | ± | 0.3 |  | 8.9 | ± | 3.6 | 4.5 | ± | 1.2 |  | 12.8 | ± | 3.3 | 4.2 | ± | 0.8 |  | 24.3 | ± | 7.0 | 4.9 | ± | 1.5 |  | 29.1 | ± | 7.6 | 15.7 | ± | 3.6 |
|  | **Neutrophils** | 0.3 | ± | 0.2 | 0.5 | ± | 0.3 |  | 25.0 | ± | 7.6 | 14.2 | ± | 3.2 |  | 77.2 | ± | 18.2 | 26.0 | ± | 3.3 |  | 168.1 | ± | 34.6 | 30.7 | ± | 4.3 |  | 54.3 | ± | 5.7 | 30.3 | ± | 3.7 |
|  | **Eosinophils** | 0.2 | ± | 0.1 | 0.3 | ± | 0.2 |  | 79.1 | ± | 19.7 | 43.7 | ± | 4.4 |  | 145.4 | ± | 35.3 | 48.9 | ± | 2.9 |  | 254.1 | ± | 13.4 | 49.4 | ± | 4.0 |  | 24.9 | ± | 6.4 | 14.0 | ± | 3.6 |
|  | **Epithelial** | 1.6 | ± | 0.4 | 2.8 | ± | 0.6 |  | 4.7 | ± | 2.6 | 2.5 | ± | 0.9 |  | 3.4 | ± | 1.1 | 1.3 | ± | 0.4 |  | 2.1 | ± | 1.3 | 0.4 | ± | 0.3 |  | 4.3 | ± | 0.8 | 2.3 | ± | 0.4 |
| **Day 28** | |  |  |  |  |  |  |  |  |  |  |  |  |  |  |  |  |  |  |  |  |  |  |  |  |  |  |  |  |  |  |  |  |  |  |
|  | **Total BAL cells** | 63.5 | ± | 10.8 | 100 |  |  |  | 57.1 | ± | 5.0 | 100 |  |  |  | 78.0 | ± | 12.5 | 100 |  |  |  | 291.7 | ± | 52.7 | 100 |  |  |  | 132.5 | ± | 15.3 | 100 |  |  |
|  | **Macrophages** | 55.7 | ± | 9.4 | 88.6 | ± | 3.8 |  | 53.0 | ± | 5.0 | 92.6 | ± | 1.3 |  | 59.3 | ± | 8.0 | 79.4 | ± | 8.0 |  | 164.2 | ± | 38.6 | 55.7 | ± | 6.6 |  | 73.6 | ± | 14.9 | 54.4 | ± | 6.6 |
|  | **Lymphocytes** | 1.5 | ± | 1.2 | 2.0 | ± | 1.5 |  | 0.6 | ± | 0.2 | 1.1 | ± | 0.4 |  | 13.5 | ± | 7.8 | 13.8 | ± | 8.2 |  | 94.4 | ± | 18.1 | 32.7 | ± | 4.5 |  | 29.3 | ± | 9.3 | 23.2 | ± | 6.4 |
|  | **Neutrophils** | 0.2 | ± | 0.1 | 0.4 | ± | 0.2 |  | 0.0 | ± | 0.0 | 0.1 | ± | 0.1 |  | 1.7 | ± | 1.0 | 1.6 | ± | 0.9 |  | 21.6 | ± | 12.7 | 7.0 | ± | 3.6 |  | 23.4 | ± | 1.4 | 18.5 | ± | 1.8 |
|  | **Eosinophils** | 2.2 | ± | 1.9 | 3.2 | ± | 2.5 |  | 0.0 | ± | 0.0 | 0.0 | ± | 0.0 |  | 0.2 | ± | 0.1 | 0.3 | ± | 0.1 |  | 6.6 | ± | 4.1 | 2.8 | ± | 2.0 |  | 0.4 | ± | 0.2 | 0.2 | ± | 0.2 |
|  | **Epithelial** | 4.0 | ± | 1.3 | 5.8 | ± | 1.0 |  | 3.5 | ± | 0.9 | 6.2 | ± | 1.5 |  | 3.3 | ± | 1.0 | 4.9 | ± | 1.6 |  | 4.9 | ± | 1.2 | 1.8 | ± | 0.4 |  | 5.8 | ± | 3.4 | 3.7 | ± | 1.6 |
| **Month 3** | |  |  |  |  |  |  |  |  |  |  |  |  |  |  |  |  |  |  |  |  |  |  |  |  |  |  |  |  |  |  |  |  |  |  |
|  | **Total BAL cells** | 60.1 | ± | 17.2 | 100 |  |  |  |  |  |  |  |  |  |  |  |  |  |  |  |  |  | 89.8 | ± | 32.7 | 100 |  |  |  | 129.9 | ± | 31.4 | 100 |  |  |
|  | **Macrophages** | 55.7 | ± | 16.3 | 91.7 | ± | 1.0 |  |  |  |  |  |  |  |  |  |  |  |  |  |  |  | 77.8 | ± | 26.8 | 88.4 | ± | 1.6 |  | 107.0 | ± | 31.9 | 79.9 | ± | 3.9 |
|  | **Lymphocytes** | 0.3 | ± | 0.2 | 0.9 | ± | 0.7 |  |  |  |  |  |  |  |  |  |  |  |  |  |  |  | 4.8 | ± | 2.4 | 4.7 | ± | 1.0 |  | 10.9 | ± | 2.3 | 10.6 | ± | 3.1 |
|  | **Neutrophils** | 1.1 | ± | 0.4 | 2.3 | ± | 1.1 |  |  |  |  |  |  |  |  |  |  |  |  |  |  |  | 0.6 | ± | 0.3 | 0.8 | ± | 0.5 |  | 7.0 | ± | 1.3 | 5.6 | ± | 0.7 |
|  | **Eosinophils** | 0.0 | ± | 0.0 | 0.1 | ± | 0.1 |  |  |  |  |  |  |  |  |  |  |  |  |  |  |  | 0.0 | ± | 0.0 | 0.0 | ± | 0.0 |  | 0.3 | ± | 0.2 | 0.3 | ± | 0.1 |
|  | **Epithelial** | 3.0 | ± | 0.9 | 5.0 | ± | 1.4 |  |  |  |  |  |  |  |  |  |  |  |  |  |  |  | 6.5 | ± | 3.5 | 6.1 | ± | 1.1 |  | 4.6 | ± | 1.5 | 3.6 | ± | 1.2 |
|  |  |  |  |  |  |  |  |  |  |  |  |  |  |  |  |  |  |  |  |  |  |  |  |  |  |  |  |  |  |  |  |  |  |  |  |
|  |  | * |  | *** |  |  |  |  |  |  |  |  |  |  |  |  |  |  |  |  |  |  |  |  |  |  |  |  |  |  |  |  |  |  |  |
|  |  | ** |  | **** |  |  |  |  |  |  |  |  |  |  |  |  |  |  |  |  |  |  |  |  |  |  |  |  |  |  |  |  |  |  |  |

Mean ± standard error of the mean. Sample size: n = 6. Statistical significance *p<0.05, **p<0.01, ***p<0.001, ****p<0.0001 compared to control.

**Table S6**. Cellular composition of bronchoalveolar lavage 1, 3, 28 days and 3 months after exposure to MWCNT Mitsui-7.

|  |  | **Vehicle control** | | | | | |  | **MWCNT Mitsui-7** | | | | | | | | | | | | | | | | | | | |
| --- | --- | --- | --- | --- | --- | --- | --- | --- | --- | --- | --- | --- | --- | --- | --- | --- | --- | --- | --- | --- | --- | --- | --- | --- | --- | --- | --- | --- |
|  |  | 0 µg | | | | | |  | 6 µg | | | | | |  | 18 µg | | | | | |  | 54 µg | | | | | |
|  |  | Cell count (x10^3^) | | |  | % |  |  | Cell count (x10^3^) | | |  | % |  |  | Cell count (x10^3^) | | |  | % |  |  | Cell count (x10^3^) | | |  | % |  |
| **Day 1** | |  |  |  |  |  |  |  |  |  |  |  |  |  |  |  |  |  |  |  |  |  |  |  |  |  |  |  |
|  | **Total BAL cells** | 54.8 | ± | 3.9 | 100 |  |  |  | 92.5 | ± | 14.9 | 100 |  |  |  | 147.7 | ± | 23.8 | 100 |  |  |  | 125.8 | ± | 10.3 | 100 |  |  |
|  | **Macrophages** | 44.1 | ± | 3.3 | 80.3 | ± | 2.4 |  | 48.6 | ± | 5.3 | 55.6 | ± | 4.1 |  | 45.1 | ± | 7.5 | 33.5 | ± | 4.5 |  | 43.0 | ± | 7.2 | 32.9 | ± | 3.5 |
|  | **Lymphocytes** | 0.6 | ± | 0.2 | 1.1 | ± | 0.3 |  | 1.3 | ± | 0.3 | 1.4 | ± | 0.1 |  | 2.9 | ± | 0.8 | 1.7 | ± | 0.4 |  | 1.4 | ± | 0.3 | 1.1 | ± | 0.3 |
|  | **Neutrophils** | 5.6 | ± | 1.5 | 9.7 | ± | 2.4 |  | 22.9 | ± | 5.3 | 23.6 | ± | 2.4 |  | 63.3 | ± | 11.8 | 41.9 | ± | 2.6 |  | 57.9 | ± | 4.2 | 48.1 | ± | 5.3 |
|  | **Eosinophils** | 0.4 | ± | 0.2 | 0.7 | ± | 0.3 |  | 15.7 | ± | 5.1 | 15.4 | ± | 2.6 |  | 31.2 | ± | 6.9 | 19.6 | ± | 3.4 |  | 14.1 | ± | 4.5 | 10.4 | ± | 3.3 |
|  | **Epithelial** | 3.9 | ± | 0.5 | 7.7 | ± | 1.2 |  | 4.0 | ± | 1.1 | 4.1 | ± | 0.5 |  | 5.1 | ± | 1.5 | 3.2 | ± | 0.6 |  | 9.2 | ± | 2.2 | 7.4 | ± | 1.7 |
| **Day 3** | |  |  |  |  |  |  |  |  |  |  |  |  |  |  |  |  |  |  |  |  |  |  |  |  |  |  |  |
|  | **Total BAL cells** | 62.9 | ± | 3.8 | 100 |  |  |  | 407.4 | ± | 108.7 | 100 |  |  |  | 308.5 | ± | 56.9 | 100 |  |  |  | 446.1 | ± | 43.6 | 100 |  |  |
|  | **Macrophages** | 53.3 | ± | 4.2 | 84.3 | ± | 2.2 |  | 75.9 | ± | 11.0 | 22.1 | ± | 2.5 |  | 41.3 | ± | 5.7 | 14.6 | ± | 1.2 |  | 88.5 | ± | 10.7 | 20.8 | ± | 2.9 |
|  | **Lymphocytes** | 0.9 | ± | 0.3 | 1.6 | ± | 0.5 |  | 16.4 | ± | 3.3 | 4.5 | ± | 0.6 |  | 9.2 | ± | 1.7 | 3.4 | ± | 0.7 |  | 40.2 | ± | 6.8 | 9.2 | ± | 1.5 |
|  | **Neutrophils** | 1.0 | ± | 0.7 | 1.9 | ± | 0.3 |  | 9.6 | ± | 4.9 | 1.9 | ± | 0.6 |  | 23.7 | ± | 5.8 | 7.7 | ± | 1.3 |  | 93.4 | ± | 16.6 | 21.6 | ± | 3.3 |
|  | **Eosinophils** | 1.3 | ± | 1.0 | 2.0 | ± | 1.5 |  | 300.2 | ± | 92.1 | 69.5 | ± | 2.9 |  | 231.2 | ± | 46.2 | 73.0 | ± | 2.2 |  | 216.9 | ± | 41.7 | 46.8 | ± | 5.3 |
|  | **Epithelial** | 6.3 | ± | 0.9 | 10.2 | ± | 1.5 |  | 5.3 | ± | 0.9 | 1.9 | ± | 0.6 |  | 2.9 | ± | 0.5 | 1.3 | ± | 0.3 |  | 6.5 | ± | 1.3 | 1.5 | ± | 0.3 |
| **Day 28** | |  |  |  |  |  |  |  |  |  |  |  |  |  |  |  |  |  |  |  |  |  |  |  |  |  |  |  |
|  | **Total BAL cells** | 62.8 | ± | 10.5 | 100 |  |  |  | 99.3 | ± | 10.3 | 100 |  |  |  | 107.8 | ± | 7.1 | 100 |  |  |  | 13.1 | ± | 17.1 | 100 |  |  |
|  | **Macrophages** | 45.7 | ± | 7.0 | 75.8 | ± | 5.4 |  | 66.1 | ± | 6.3 | 68.4 | ± | 4.5 |  | 51.2 | ± | 13.7 | 48.9 | ± | 13.0 | | 89.5 | ± | 8.6 | 71.2 | ± | 4.9 |
|  | **Lymphocytes** | 4.1 | ± | 1.5 | 5.4 | ± | 1.9 |  | 10.0 | ± | 2.6 | 9.2 | ± | 2.2 |  | 9.1 | ± | 3.7 | 8.3 | ± | 3.1 |  | 12.3 | ± | 4.6 | 8.2 | ± | 2.2 |
|  | **Neutrophils** | 1.6 | ± | 1.0 | 1.8 | ± | 1.0 |  | 6.9 | ± | 1.5 | 6.6 | ± | 1.2 |  | 5.6 | ± | 1.8 | 5.5 | ± | 1.9 |  | 10.3 | ± | 2.9 | 7.3 | ± | 1.9 |
|  | **Eosinophils** | 3.1 | ± | 2.7 | 4.9 | ± | 4.2 |  | 11.6 | ± | 3.5 | 11.1 | ± | 3.3 |  | 7.1 | ± | 2.5 | 6.5 | ± | 2.2 |  | 12.3 | ± | 6.7 | 7.6 | ± | 3.9 |
|  | **Epithelial** | 8.2 | ± | 2.4 | 12.2 | ± | 2.5 |  | 4.8 | ± | 0.8 | 4.6 | ± | 0.6 |  | 2.2 | ± | 0.8 | 2.2 | ± | 0.9 |  | 6.5 | ± | 1.3 | 5.6 | ± | 1.6 |
| **Month 3** | |  |  |  |  |  |  |  |  |  |  |  |  |  |  |  |  |  |  |  |  |  |  |  |  |  |  |  |
|  | **Total BAL cells** | 55.9 | ± | 7.1 | 100 |  |  |  | 50.2 | ± | 6.0 | 100 |  |  |  | 59.4 | ± | 8.5 | 100 |  |  |  | 99.4 | ± | 6.8 | 100 |  |  |
|  | **Macrophages** | 45.9 | ± | 5.4 | 83.1 | ± | 2.4 |  | 41.5 | ± | 5.0 | 82.3 | ± | 3.9 |  | 51.0 | ± | 7.6 | 85.1 | ± | 2.7 |  | 90.4 | ± | 6.3 | 90.9 | ± | 1.0 |
|  | **Lymphocytes** | 3.2 | ± | 1.6 | 5.1 | ± | 2.1 |  | 2.6 | ± | 1.6 | 4.3 | ± | 2.1 |  | 2.1 | ± | 0.5 | 3.1 | ± | 0.7 |  | 2.6 | ± | 0.7 | 2.6 | ± | 0.8 |
|  | **Neutrophils** | 1.6 | ± | 0.1 | 2.8 | ± | 0.8 |  | 1.5 | ± | 0.4 | 3.1 | ± | 0.6 |  | 2.2 | ± | 0.5 | 3.4 | ± | 0.5 |  | 2.3 | ± | 0.5 | 2.2 | ± | 0.4 |
|  | **Eosinophils** | 0.0 | ± | 0.0 | 0.2 | ± | 0.1 |  | 1.0 | ± | 0.5 | 1.8 | ± | 0.8 |  | 0.6 | ± | 0.3 | 1.0 | ± | 0.5 |  | 0.7 | ± | 0.3 | 0.7 | ± | 0.3 |
|  | **Epithelial** | 5.1 | ± | 1.0 | 8.7 | ± | 0.9 |  | 3.6 | ± | 1.0 | 8.6 | ± | 3.5 |  | 3.5 | ± | 1.3 | 7.4 | ± | 3.2 |  | 3.4 | ± | 0.7 | 3.4 | ± | 0.7 |
|  |  |  |  |  |  |  |  |  |  |  |  |  |  |  |  |  |  |  |  |  |  |  |  |  |  |  |  |  |
|  |  | * |  | *** |  |  |  |  |  |  |  |  |  |  |  |  |  |  |  |  |  |  |  |  |  |  |  |  |
|  |  | ** |  | **** |  |  |  |  |  |  |  |  |  |  |  |  |  |  |  |  |  |  |  |  |  |  |  |  |

Mean ± standard error of the mean. Sample size: n = 7-9. Statistical significance *p<0.05, **p<0.01, ***p<0.001, ****p<0.0001 compared to control.

## Genotoxicity in BAL cells, lung and liver in 3-month study

Genotoxicity was assessed in terms of DNA strand break levels in the comet assay measured as % DNA in the tail (Jackson, Pedersen et al. 2013). The Comet assay is a sensitive, but unspecific measure of genotoxicity as it detects abasic sites and strand breaks in the DNA. Increased levels of DNA strand breaks is interpreted as increased genotoxicity, while decreased levels of DNA strand breaks are more difficult to interpret. Lagging strand synthesis during DNA synthesis contributes to background levels of DNA strand breaks. It is therefore possible that treatment-induced cytotoxicity or lowered rate of cell division will lead to lower levels of DNA strand breaks. Consequently, we cannot meaningfully interpret lowered DNA strand break levels.


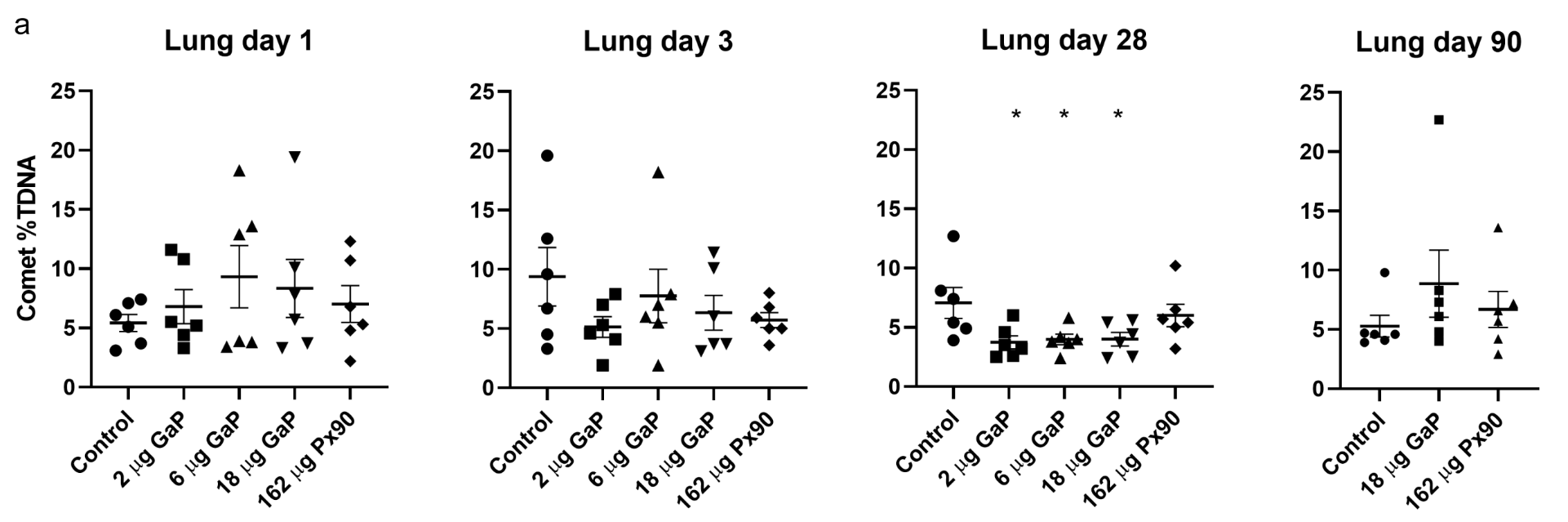

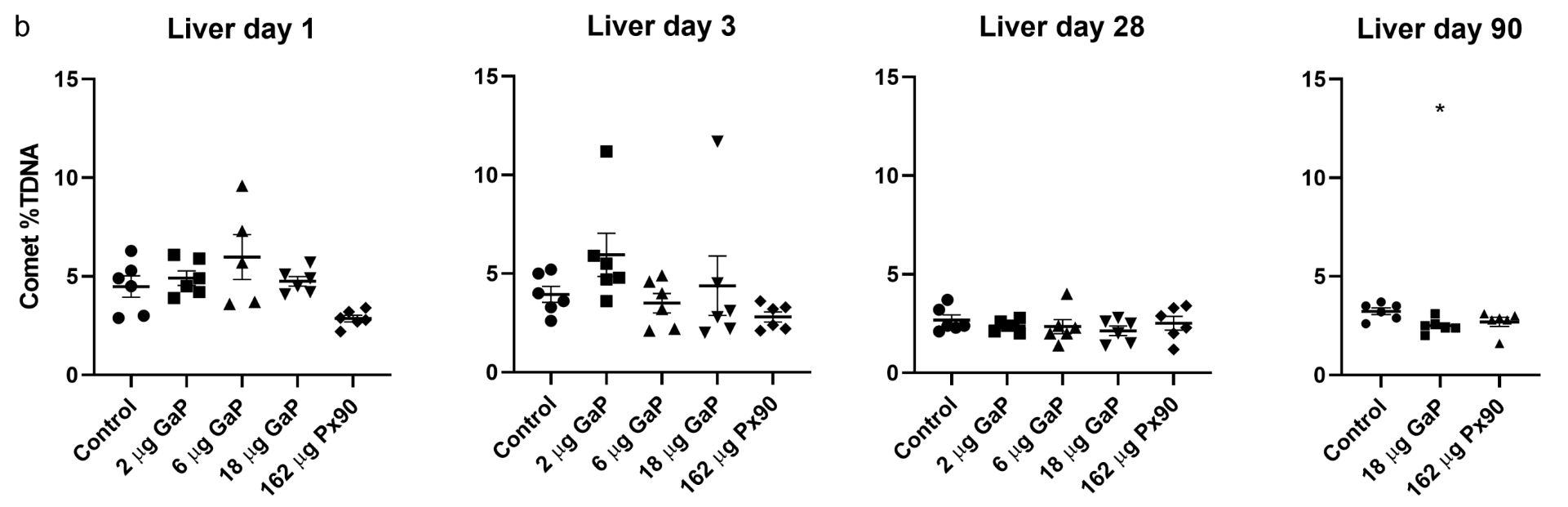


**Figure S7**. DNA strand breaks in a) lung and b) liver tissue 1, 3, 28 days and 3 months post-exposure. %TDNA: percent tail DNA. Dot plot with mean ± standard error of the mean. Sample size: n = 6. Statistical significance *p<0.05.

**Table S7.** Genotoxicity in BAL cells, lung and liver tissue in 3-month study

|  |  | **Vehicle control** | | |  | **GaP NW** | | | | | | | | | | |  | **CB** | | |
| --- | --- | --- | --- | --- | --- | --- | --- | --- | --- | --- | --- | --- | --- | --- | --- | --- | --- | --- | --- | --- |
|  |  | 0 µg | | |  | 2 µg | | |  | 6 µg | | |  | 18 µg | | |  | 162 µg | | |
| **Day 1** | |  |  |  |  |  |  |  |  |  |  |  |  |  |  |  |  |  |  |  |
|  | **BAL TL** | 15.74 | ± | 1.09 |  | 14.83 | ± | 1.05 |  | 18.53 | ± | 1.25 |  | 17.97 | ± | 1.11 |  | 13.96 | ± | 0.60 |
|  | **BAL %TDNA** | 4.78 | ± | 0.65 |  | 4.20 | ± | 0.43 |  | 6.98 | ± | 0.81 |  | 7.60 | ± | 0.96 * |  | 5.30 | ± | 0.33 |
|  | **Lung TL** | 17.52 | ± | 1.16 |  | 19.53 | ± | 2.43 |  | 23.75 | ± | 3.85 |  | 21.12 | ± | 2.50 |  | 19.71 | ± | 2.47 |
|  | **Lung %TDNA** | 5.42 | ± | 0.72 |  | 6.80 | ± | 1.43 |  | 9.32 | ± | 2.63 |  | 8.33 | ± | 2.45 |  | 7.02 | ± | 1.56 |
|  | **Liver TL** | 16.54 | ± | 1.45 |  | 17.32 | ± | 0.81 |  | 16.26 | ± | 0.84 |  | 17.12 | ± | 1.46 |  | 11.99 | ± | 0.47 * |
|  | **Liver %TDNA** | 4.48 | ± | 0.54 |  | 4.92 | ± | 0.37 |  | 5.98 | ± | 1.14 |  | 4.75 | ± | 0.25 |  | 2.87 | ± | 0.17 |
| **Day 3** | |  |  |  |  |  |  |  |  |  |  |  |  |  |  |  |  |  |  |  |
|  | **BAL TL** | 12.72 | ± | 1.00 |  | 18.69 | ± | 1.52 ** |  | 19.10 | ± | 0.71 ** |  | 19.44 | ± | 1.22 ** |  | 16.20 | ± | 1.20 |
|  | **BAL %TDNA** | 3.35 | ± | 0.54 |  | 9.52 | ± | 1.15 **** |  | 10.88 | ± | 0.68 **** |  | 11.03 | ± | 0.88 **** |  | 7.00 | ± | 0.61 * |
|  | **Lung TL** | 24.10 | ± | 3.66 |  | 16.60 | ± | 1.28 |  | 20.07 | ± | 3.23 |  | 19.04 | ± | 2.08 |  | 18.74 | ± | 1.20 |
|  | **Lung %TDNA** | 9.38 | ± | 2.47 |  | 5.13 | ± | 0.88 |  | 7.75 | ± | 2.25 |  | 6.33 | ± | 1.46 |  | 5.72 | ± | 0.63 |
|  | **Liver TL** | 14.48 | ± | 1.57 |  | 15.62 | ± | 0.95 |  | 14.11 | ± | 2.61 |  | 14.16 | ± | 3.56 |  | 12.23 | ± | 0.66 |
|  | **Liver %TDNA** | 3.95 | ± | 0.41 |  | 5.95 | ± | 1.10 |  | 3.50 | ± | 0.49 |  | 4.38 | ± | 1.51 |  | 2.80 | ± | 0.26 |
| **Day 28** | |  |  |  |  |  |  |  |  |  |  |  |  |  |  |  |  |  |  |  |
|  | **BAL TL** | 13.51 | ± | 0.91 |  | 12.15 | ± | 0.51 |  | 14.08 | ± | 1.01 |  | 14.57 | ± | 1.05 |  | 11.54 | ± | 0.30 |
|  | **BAL %TDNA** | 3.86 | ± | 0.60 |  | 2.38 | ± | 0.31 |  | 3.88 | ± | 0.84 |  | 3.17 | ± | 0.18 |  | 3.40 | ± | 0.26 |
|  | **Lung TL** | 20.76 | ± | 1.95 |  | 15.51 | ± | 0.76 * |  | 14.44 | ± | 0.32 ** |  | 14.82 | ± | 1.01 ** |  | 18.47 | ± | 1.49 |
|  | **Lung %TDNA** | 7.07 | ± | 1.30 |  | 3.73 | ± | 0.55 * |  | 3.98 | ± | 0.45 * |  | 4.00 | ± | 0.57 * |  | 6.00 | ± | 0.95 |
|  | **Liver TL** | 11.12 | ± | 0.84 |  | 9.09 | ± | 0.32 |  | 8.85 | ± | 0.38 * |  | 9.47 | ± | 0.66 |  | 13.44 | ± | 0.55 * |
|  | **Liver %TDNA** | 2.68 | ± | 0.25 |  | 2.38 | ± | 0.12 |  | 2.35 | ± | 0.36 |  | 2.13 | ± | 0.24 |  | 2.52 | ± | 0.35 |
| **Month 3** | |  |  |  |  |  |  |  |  |  |  |  |  |  |  |  |  |  |  |  |
|  | **BAL TL** | 15.62 | ± | 1.44 |  |  |  |  |  |  |  |  |  | 12.68 | ± | 0.86 |  | 12.73 | ± | 1.03 |
|  | **BAL %TDNA** | 4.48 | ± | 0.50 |  |  |  |  |  |  |  |  |  | 2.70 | ± | 0.61 |  | 3.43 | ± | 0.63 |
|  | **Lung TL** | 18.00 | ± | 1.49 |  |  |  |  |  |  |  |  |  | 22.02 | ± | 3.77 |  | 18.37 | ± | 1.94 |
|  | **Lung %TDNA** | 5.28 | ± | 0.91 |  |  |  |  |  |  |  |  |  | 8.87 | ± | 2.84 |  | 6.70 | ± | 1.52 |
|  | **Liver TL** | 13.03 | ± | 0.66 |  |  |  |  |  |  |  |  |  | 9.66 | ± | 0.46 *** |  | 13.87 | ± | 0.27 |
|  | **Liver %TDNA** | 3.23 | ± | 0.17 |  |  |  |  |  |  |  |  |  | 2.50 | ± | 0.15 * |  | 2.70 | ± | 0.23 |
|  |  |  |  |  |  |  |  |  |  |  |  |  |  |  |  |  |  |  |  |  |
|  | **Increase** |  |  |  |  |  |  |  |  |  |  |  |  |  |  |  |  |  |  |  |
|  | **Decrease** |  |  |  |  |  |  |  |  |  |  |  |  |  |  |  |  |  |  |  |

Mean ± standard error of the mean. Sample size: n = 6. Statistical significance *p<0.05, **p<0.01, ***p<0.001, ****p<0.0001.

## **Table S8. Mouse lung histopathology 1, 28 days and 3 months after intratracheal instillation of GaP NWs. Incidence table. Group size = 3-8.**

|  | Group | Lymphocytic infiltrates | | Macrophage aggregates | | Eosinophilic infiltrates | | Proteinosis | Giant cell |
| --- | --- | --- | --- | --- | --- | --- | --- | --- | --- |
|  |  | Mean^a^ | Incidence | Mean^a^ | Incidence | Mean^a^ | Incidence |  |  |
| Day 1 | |  |  |  |  |  |  |  |  |
|  | Vehicle control | 0.0 | 0/6 | 0.0 | 0/6 | 0.3 | 2/6 | 0/6 | 0/6 |
|  | GaP NW 3 µg | 0.0 | 0/3 | 1.0 | 2/3 | 1.0 | 2/3 | 0/3 | 0/3 |
|  | GaP NW 6 µg | 0.0 | 0/3 | 2.0 | 3/3 | 2.7 | 3/3 | 0/3 | 0/3 |
|  | GaP NW 18 µg | 0.0 | 0/6 | 0.9 | 3/6 | 2.1 | 6/6 | 0/6 | 0/6 |
|  |  |  |  |  |  |  |  |  |  |
| Day 28 | |  |  |  |  |  |  |  |  |
|  | Vehicle control | 0.3 | 2/8 | 0.0 | 0/8 | 0.0 | 0/8 | 0/8 | 0/8 |
|  | GaP NW 3 µg | 0.0 | 0/5 | 0.0 | 0/5 | 0.0 | 0/5 | 0/5 | 1/5 |
|  | GaP NW 6 µg | 0.0 | 0/5 | 0.4 | 2/5 | 0.0 | 0/5 | 3/5 | 0/5 |
|  | GaP NW 18 µg | 0.0 | 0/8 | ^b^ | 8/8 | 0.0 | 0/8 | 8/8 | 8/8 |
|  |  |  |  |  |  |  |  |  |  |
| Month 3 | |  |  |  |  |  |  |  |  |
|  | Vehicle control | 0.1 | 1/8 | 0.0 | 0/8 | 0.0 | 0/8 | 0/8 | 0/8 |
|  | GaP NW 18 µg ^c^ | 0.0 | 0/6 | 0.5 | 3/6 | 0.0 | 0/6 | 0/6 | 0/6 |

^a^ Mean number of lymphocytic infiltrates (minimum 50 lymphocytes)/macrophage aggregates (minimum 5 macrophages)/eosinophilic infiltrates (minimum 20 eosinophils) counted in the lung section with most histopathological changes. ^b^ One macrophage aggregate (4/8), diffuse macrophage infiltration covering 5% (3/8) or 30% of lung lobe (1/8). ^c^ NWs in macrophages were observed in all animals (6/6) 3 months post-exposure. One animal with interstitial thickening (1/6) at month 3.

## **Table S9. Composition of 0.02M potassium hydrogen phthalate buffered phagolysosomal simulant fluid (PSF), pH 4.5**.

| Component | Chemical Formula | Concentration [mg/L] |
| --- | --- | --- |
| Sodium phosphate dibasic anhydrous | Na_2_HPO_4_ | 142 |
| Sodium chloride | NaCl | 6650 |
| Sodium sulfate anhydrous | Na_2_SO_4_ | 71 |
| Calcium chloride dihydrate | CaCl_2_·2H_2_O | 29 |
| Glycine | H_2_NCH_2_CO_2_H | 450 |
| Potassium hydrogen phthalate | (1-(HO_2_C)-2-(CO_2_K)-C_6_H_4_) | 4085 |
| Alkylbenzyldimethylammonium chloride | - | 50 ppm |

## Table S10. Composition of low-calcium Gamble’s solution, pH 7.4 adjusted by HCl.

| Component | Chemical Formula | Concentration [mg/L] |
| --- | --- | --- |
| Sodium chloride | NaCl | 6600 |
| Sodium bicarbonate | NaHCO_3_ | 2703 |
| Calcium chloride | CaCl_2_ | 22 |
| Sodium phosphate dibasic dodecahydrate | Na_2_HPO_4_·12H_2_O | 358 |
| Sodium sulfate anhydrous | Na_2_SO_4_ | 79 |
| Magnesium chloride hexahydrate | MgCl·6H_2_O | 212 |
| Glycine | H_2_NCH_2_CO_2_H | 118 |
| Sodium citrate dihydrate | Na_3_C_6_H_5_O_7_·2H_2_O | 153 |
| Sodium tartrate dihydrate | Na_2_C_4_H_4_O_6_·2H_2_O | 180 |
| Sodium pyruvate | C_3_H_3_NaO_3_ | 172 |
| Sodium lactate | C_3_H_3_NaO_3_ | 175 |

# References

Jackson, P., L. M. Pedersen, Z. O. Kyjovska, N. R. Jacobsen, A. T. Saber, K. S. Hougaard, U. Vogel and H. Wallin (2013). "Validation of freezing tissues and cells for analysis of DNA strand break levels by comet assay." Mutagenesis **28**(6): 699-707.
